# ES1 is a mitochondrial enlarging factor contributing to form mega-mitochondria in zebrafish cones

**DOI:** 10.1038/srep22360

**Published:** 2016-03-01

**Authors:** Takamasa Masuda, Yasutaka Wada, Satoru Kawamura

**Affiliations:** 1 Graduate School of Frontier Biosciences, Osaka University, Suita, Osaka 565-0871, Japan; 2 Department of Biological Sciences, Graduate School of Science, Osaka University, Suita, Osaka 565-0871, Japan

## Abstract

Total mass of mitochondria increases during cell proliferation and differentiation through mitochondrial biogenesis, which includes mitochondrial proliferation and growth. During the mitochondrial growth, individual mitochondria have been considered to be enlarged independently of mitochondrial fusion. However, molecular basis for this enlarging process has been poorly understood. Cone photoreceptor cells in the retina possess large mitochondria, so-called mega-mitochondria that have been considered to arise via the enlarging process. Here we show that ES1 is a novel mitochondria-enlarging factor contributing to form mega-mitochondria in cones. ES1 is specifically expressed in cones and localized to mitochondria including mega-mitochondria. Knockdown of ES1 markedly reduced the mitochondrial size in cones. In contrast, ectopic expression of ES1 in rods significantly increased both the size of individual mitochondria and the total mass of the mitochondrial cluster without changing the number of them. RNA-seq analysis showed that ERRα and its downstream mitochondrial genes were significantly up-regulated in the ES1-expressing rods, suggesting facilitation of mitochondrial enlargement via ERRα-dependent processes. Furthermore, higher energy state was detected in the ES1-expressing rods, indicating that the enlarged mitochondria by ES1 are capable of producing high energy. ES1 is the mitochondrial protein that is first found to promote enlargement of individual mitochondria.

Both rod and cone photoreceptor cells are high energy-consuming cells^1^,^2^,^3^. To meet this high energy demand, numerous mitochondria cluster in the photoreceptor ellipsoid adjacent to the outer segment in which light signals are converted to membrane potential changes. Cones have been considered to require more energy than rods^4^. Accordingly, cones possess higher total mitochondrial mass than rods in many vertebrate species^5^,^6^. In addition, extremely large mitochondria, mega-mitochondria, exceeding 2 μm in diameter are observed in cones of certain species such as shrew and zebrafish^7^,^8^,^9^,^10^. In a developing retina, a single mega-mitochondrion has been suggested to be formed by enlargement of a single mitochondrion^10^, not by the fusion of small mitochondria. To contribute to the study on the mitochondria biogenesis, a fusion-independent enlarging process of mitochondria, we focused on the formation of mitochondria in cones.

A 31 kDa protein, ES1, was found abundantly in the zebrafish retina and specifically in its cone ellipsoids^11^. ES1 has been predicted to be localized to mitochondria because of a putative mitochondrial localization signal (MLS) present at the N-terminus^12^. Orthologues of ES1 are conserved across species from *E. coli* (σ cross-reacting protein 27A) to human, with strong similarities ranging from 77 to 81%, implying their significant physiological role(s) conserved among prokaryote and eukaryotic mitochondria^13^. These facts together with a previous finding in our laboratory that ES1 is highly abundant compared with any other proteins in purified carp cones consisting of outer segment plus ellipsoid (see Fig. 2D in ref. 14) led us to investigate the physiological role(s) of ES1 in zebrafish cone mitochondria.

In the present study, we clearly showed that ES1 is a mitochondria-enlarging factor and contributes to form mega-mitochondria in cones. Furthermore, our data strongly suggested that ES1 supports energy production via mitochondrial enlargement.

## Results

### ES1 is localized in cone mitochondria

**Figure 1:**
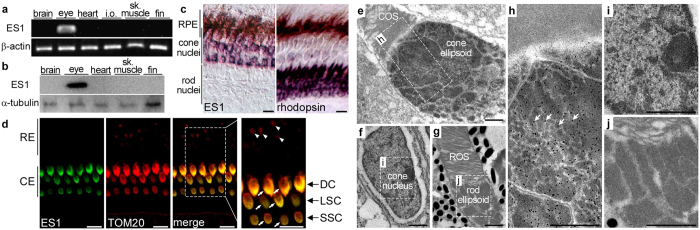
ES1 is specifically expressed in cones and localized to mitochondria. (**a**) RT-PCR assay for ES1 gene expression in zebrafish tissues. Beta-actin was used as an internal control for a constant amount of RNA templates. i.o. internal organs, sk. muscle: skeletal muscle. (**b**) Immunoblot assay with ES1 antibody in zebrafish tissues. Alpha-tubulin antibody was used as a loading control. sk. muscle: skeletal muscle. (**c**) *In situ* hybridization on adult zebrafish retina. ES1 mRNA was only detected around cone nuclei (purple staining, left panel). Rhodopsin probe was used as a rod maker (right panel). Dark-brown staining in RPE (retinal pigment epithelium) layer is due to endogenous melanin pigment. Scale bars, 10 μm. (**d**) Immunohistochemistry on adult zebrafish retina with ES1 (green) and TOM20 (mitochondrial marker, red) antibodies. ES1-immunoreactivity was detected in ellipsoids in all types of cones (i.e., double, long-single and short-single cones, arrows) but not in rod ellipsoids (arrow heads). Scale bars, 10 μm. RE: rod ellipsoid, CE: cone ellipsoid, DC: double cone, LSC: long-single cone, SSC: short-single cone. (**e**–**j**) Immuno-gold electron microscopy with ES1 antibody on adult zebrafish retina. Each panel shows a cone ellipsoid with an outer segment (in **e**), a cone nucleus (in **f**) or a rod ellipsoid with an outer segment (in **g**). Panel (**h–j)** are magnified views of the areas surrounded by dashed lines in (**e**–**g**), respectively. A mega-mitochondrion was observed in the apical region of the cone ellipsoid (in (**e)** and (**h**), adjacent to the outer segment). Arrows indicate gold particles in (**h**). Scale bars, 1 μm in (**e**–**j**). COS: cone outer segment, ROS: rod outer segment.

In an attempt to elucidate the physiological role(s) of ES1, we firstly investigated the spatial distribution of ES1 in zebrafish. Both RT-PCR (Fig. 1a) and immunoblot analyses (Fig. 1b) using specific antibodies (Supplementary Fig. S1) demonstrated that expression of ES1 is restricted to the eye, and *in situ* hybridization analysis revealed that ES1 mRNA is expressed only in cones in the eye (Fig. 1c). ES1-immunoreactivities were specifically detected in cone ellipsoids and were co-localized with translocase of outer mitochondrial membrane 20 (TOM20), indicating specific localization of ES1 to mitochondria (Fig. 1d). Immunoelectron microscopy also demonstrated mitochondria-specific localization of ES1 (Fig. 1e–j). ES1-immunopositive signals were uniformly distributed throughout all mitochondria in cone ellipsoids including mega-mitochondria (Fig. 1e,h). However, at higher magnification under a light microscope, ES1-immunopositive signals were weaker at apical (adjacent to the outer segment) and central regions of the cone ellipsoids where mega-mitochondria are located (Supplementary Fig. S2). This is probably due to low antibody permeability to access the inside of mega-mitochondria because similar immunostaining pattern was observed with antibody against mitochondrial aspartate aminotransferase (mAAT), a mitochondrial matrix-marker protein (Supplementary Fig. S2). Immunoblotting analysis after subcellular fractionation showed that ES1 is present in a soluble fraction but not a membrane fraction, indicating ES1-sub-localization in mitochondrial matrix and/or intermembrane space (Supplementary Fig. S2).

### ES1 knockdown reduced mitochondrial size in cones

**Figure 2:**
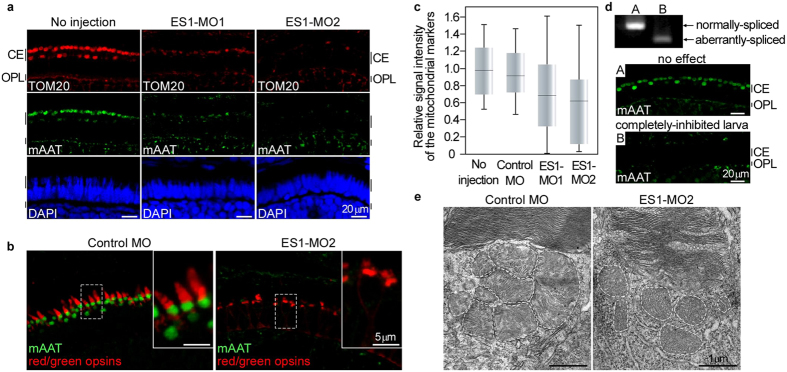
Knocking-down of ES1 resulted in formation of smaller mitochondria in cones. (**a**) Immunohistochemistry of retinal sections from ES1-MOs-injected or non-injected larvae at 4 dpf stage with both TOM20 (red) and mAAT (green) antibodies. Nuclei were stained with DAPI (blue). CE: cone ellipsoid, OPL: outer plexiform layer. (**b**) Immunohistochemistry with red/green opsins (cone outer segment marker, red) and mAAT (green) antibodies. The inset in each panel is a magnified view of the area surrounded by the dashed line. (**c**) Quantification of relative signal intensities of the mitochondrial markers from the cone ellipsoid layer of each MO-injected class; no injection (n = 28), control MO (n = 23), ES1-MO1 (n = 37) and ES1-MO2 (n = 23). Data are presented as box-whisker plots showing the median, quartiles and range. Mean value of no injection class was set to 1.0. (**d**) A paired RT-PCR (top) and immunohistochemical analysis (middle and bottom) in a single larva using a single eye for each analysis without (A) or with (B) the knock down effect of ES1-MO1. A completely-inhibited larva showed the relative immunopositive signal intensity of 0.20 (B), whereas a larva representing no inhibition showed 0.77 (A). (**e**) Representatives of electron microscopic images of cone mitochondria of control MO-injected (left panel) or ES1-MO2-injected (right panel) larvae at 4 dpf. Dashed lines outline each mitochondrion. The images without the outlines are shown in Supplementary Fig. S5.

To elucidate the physiological role(s) of ES1, we undertook the knockdown approach using two types of morpholinos (MOs) against ES1 transcript, namely ES1-MO1 and ES1-MO2 (Supplementary Fig. S3). ES1-MO1 was designed to form a frame-shifted mRNA by inhibiting a normal splicing and used to estimate the inhibition efficiency, and ES1-MO2 was designed to suppress translation. The ES1-MO1-mediated splicing inhibition was confirmed by RT-PCR in larvae at 4 days post-fertilization (dpf) stage (Supplementary Fig. S3). Eye size of ES1-MOs-injected larvae was slightly decreased compared with that of control-MO-injected larvae, suggesting that ES1 depletion affected eye growth in early stages (Supplementary Fig. S4). Any other obvious differences were not observed in whole body shape or retinal structure between the ES1-MOs and control-MO-injected larvae (Supplementary Fig. S4). In some population of larvae injected with either of the two ES1-MOs but not a control MO, immunoreactivities for two mitochondrial marker proteins, i.e., TOM20 and mAAT, were significantly reduced in the cone ellipsoid layer (Fig. 2a,b). This phenotype was not due to the loss of the ability to differentiate into cones because red/green opsins antibody gave positive signals, confirming the differentiation into cones (Fig. 2b). Quantitative analyses of the immunoreactivities to the mitochondrial markers (Fig. 2c) showed that as an average, signal intensity was reduced in the ES1-MO1-injected group. Values of the signal intensities were scattered in a wide range including the range in the control MO-injected group, and the population of the larvae showing weaker signals than the control group was 35% in the ES1-MO1-injected group. This proportion well agreed with the portion of the larvae in which normal splicing of ES1 transcript was completely suppressed (39%, Supplementary Fig. S3). Such an effect of ES1-MO1 on cone mitochondria was demonstrated more directly by a paired RT-PCR and immunohistochemical analysis in a single larva using a single eye for each measurement: a completely knocked-down larva (larva B in Fig. 2d) showed only weak immunoreactivities to the mitochondria markers in the cone ellipsoid layer (the relative signal intensity was 0.2). ES1-MO2 induced the same phenotype more effectively (Fig. 2a,c), excluding the possibility of off-target effects. To evaluate morphological changes of mitochondria, we measured the size of individual mitochondria in cone ellipsoids of the ES1-MOs-injected larvae by electron microscopy. Compared with the control MO-injected larvae, smaller mitochondria were observed in the cone ellipsoid of ES1-MO2-injected larvae (Fig. 2e), and the same phenotype was also observed in the ES1-MO1-injected larvae (Supplementary Fig. S5). These results revealed that ES1 is necessary to form large mitochondria in cones, and led us to hypothesize that ES1 contributes to enlargement of mitochondria.

### Ectopic expression of ES1 induced mitochondrial enlargement in rods

**Figure 3:**
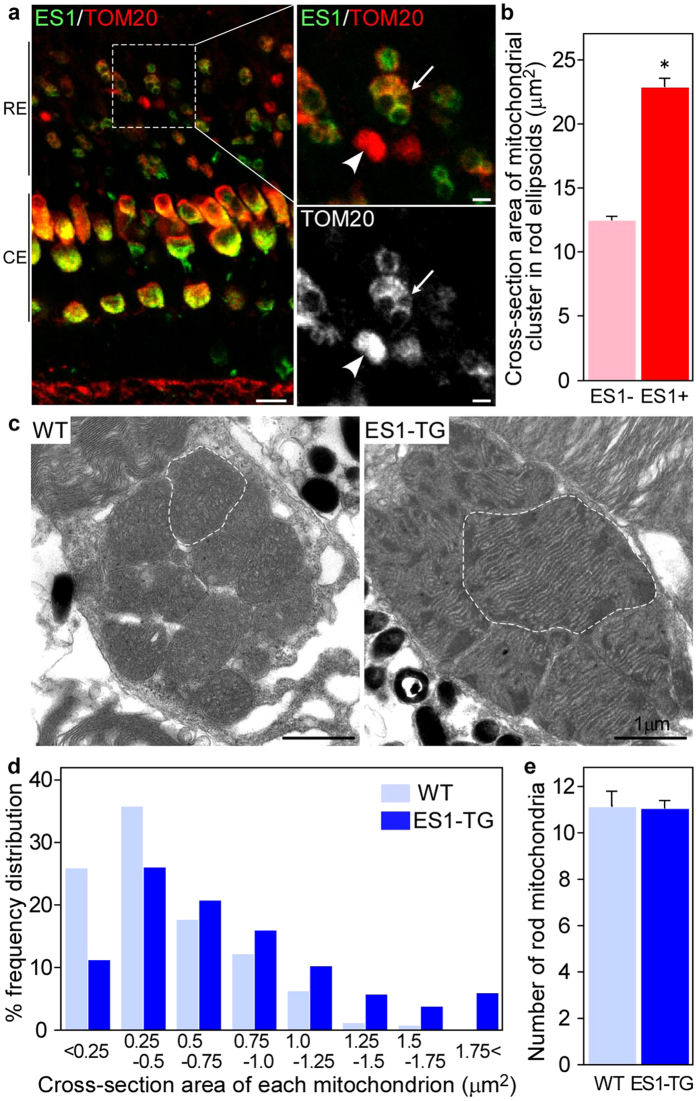
Ectopic expression of ES1 led mitochondrial enlargement in rods. (**a**) Immunohistochemistry of retinal sections from adult ES1-TG zebrafish (F_0_) with ES1 (green) and TOM20 (red) antibodies. Images are at a certain depth of confocal view. Right panels show a magnified view of merged image (upper) or TOM20 alone (lower). An arrow in each panel indicates a mitochondrial cluster in an ES1-expressing rod ellipsoid and an arrowhead indicates a mitochondrial cluster in a wild-type rod ellipsoid. As in the cone ellipsoids, immunopositive signals were weaker in the round patch in the ES1-expressing rods than the wild-type, presumably because of low antibody permeability of mega-mitochondria. Scale bars, 10 μm (left panel) and 2 μm (right panels). RE: rod ellipsoid, CE: cone ellipsoid. (**b**) Quantification of cross-section areas of mitochondrial clusters in rod ellipsoids. TOM20-immunopositive cross-section areas including immunonegative round areas were measured. Values are means ± S.E., *P = 2 × 10^−31^ in Student’s t-test, n = 120 for wild-type rods (ES1-) and n = 142 for ES1-expressing rods (ES1+). (**c**) Representatives of electron microscopic images of mitochondria in wild-type (WT) rods (left panel) or ES1-TG rods (right panel). F_1_ generation of ES1-TG zebrafish, in which all rods express ES1, was used. Dashed lines outline the largest mitochondrion in each view. (**d**) Mitochondrial size distribution in ES1-TG and WT rods. Cross-section area of each mitochondrion was measured in the electron microscopic images. n = 256 for WT and n = 420 for ES1-TG. (**e**) The number of mitochondria present in each rod. Values are means ± S.E., n = 23 for WT and n = 38 for ES1-TG.

To test the hypothesis above, we next generated transgenic (TG) zebrafish to undertake a gain-of-function analysis of ES1. ES1 was ectopically expressed in rods together with EGFP as a reporter (Supplementary Fig. S6). ES1-immunoreactivities were successfully detected in the ellipsoid of roughly a half of rods in an F_0_ mosaic ES1-TG fish (Fig. 3a left panel and Supplementary Fig. S6). Macroscopically, retinal structure was not changed in the ES1-TG fish, and cell shape of ES1-expressing rods was not changed except for the size of ellipsoids (Supplementary Fig. S6). Cross-section areas of mitochondrial clusters in the ES1-positive rod ellipsoids (green) were approximately two times larger than those in the wild-type rods (red) (Fig. 3b), indicating that the total mitochondrial mass in each rod was increased by expression of ES1. Such expansion of mitochondrial cluster was not observed in rods expressing EGFP alone or MLS-possessing EGFP (Supplementary Fig. S6). Immunostaining for TOM20 in the ES1-positive rod ellipsoids showed appearance similar to that in the cone ellipsoids: immunonegative round patches >2 μm in diameter were always observed (compare Fig. 3a right panels and panel **a** in Supplementary Fig. S2). In contrast, no such immunonegative signals were observed in the wild-type rod ellipsoids (Fig. 3a). Because TOM20 antibody immunostains the outer membrane of mitochondria^15^, such a difference in immunostaining pattern suggested enlargement of individual mitochondria in the ES1-positive rod ellipsoids. Indeed, size distribution of individual mitochondria was shifted to larger values in the electron-microscopic images of ES1-TG rods (Fig. 3c,d). Furthermore, mega-mitochondria, exceeding 2 μm in diameter, were observed in the ES1-TG rods, whereas no such huge mitochondria were observed in wild-type rods (Fig. 3c). Although the cristal membranes appears to be well aligned in the ES1-TG rod than the WT rod (Fig. 3c), significant difference was not observed in other images, suggesting that the structural variation is not related to the ES1 expression. In terms of the number of mitochondria, no significant difference was observed in each rod ellipsoid between the ES1-TG and the wild-type (Fig. 3e), indicating that ES1 induced enlargement of individual mitochondria but not mitochondrial proliferation.

### Mitochondrial biogenesis-related transcription factors were up-regulated in the ES1-expressing rods

**Figure 4:**
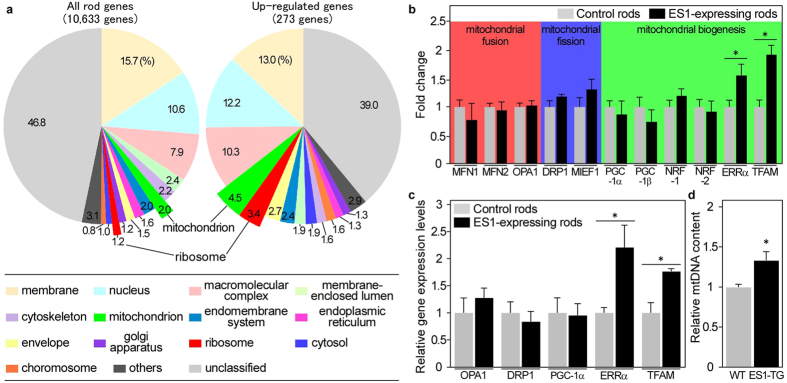
Gene expression changes associated with ectopic expression of ES1 in rods. (**a**) Composition of cellular component category based on GO slim classification for all genes detected from the ES1 and/or EGFP-expressing rods (left) and >1.5 fold up-regulated genes in the ES1-expressing rods (right). Proportion of genes classified into ribosome and mitochondrion categories were remarkably increased in the up-regulated genes. (**b**) Relative gene expression levels of mitochondrial fusion, fission and biogenesis-related genes in the ES1-expressing rods compared with those in the EGFP-expressing rods as a control. Abbreviations: MFN; mitofusin, OPA; optic atrophy, DRP; dynamin-related protein, MIEF; mitochondrial elongation factor, PGC; proliferator activated receptor gamma coactivator, NRF; nuclear respiratory factor, ERR; estrogen-related receptor, TFAM; mitochondrial transcription factor A. Values are means ± S.E. calculated from Cufflinks results for each replicate. *P < 0.05 adjusted by Benjamini and Hochberg procedure, n = 3 for both control and ES1-expressing rods. (**c**) Validation of relative expression levels of essential genes for mitochondrial fusion, fission and biogenesis by real-time PCR. Values are means ± S.E., *P < 0.05 in Student’s t-test, n = 3 for both control and ES1-expressing rods. (**d**) Quantification of mtDNA by real-time PCR in rods purified from ES1-TG or wild-type siblings. Values are means ± S.E., *P = 0.04 in Student’s t-test, n = 3 for both wild-type and ES1-expressing rods.

To elucidate the molecular mechanisms underlying the mitochondrial enlargement induced by ES1, we investigated gene expression in the ES1-expressing rods. Rods were isolated from both the ES1-TG and EGFP-TG (as the reference) retinas and purified by fluorescence-activated cell sorting (FACS). The obtained rod fractions containing 96.9 ± 1.02% rods (Supplementary Fig. S7) were then subjected to high-throughput RNA sequencing, so-called RNA-seq. Statistical analyses of the RNA-seq data revealed that 236 genes were significantly up-regulated (Supplementary Table S1) and 229 genes were down-regulated (Supplementary Table S2) in the ES1-expressing rods. Gene ontology (GO) and pathway enrichment analyses demonstrated that genes classified into cytosolic ribosomal proteins were mostly up-regulated (Fig. 4a, Table 1 and Supplementary Fig. S8), implying enhanced protein synthesis in the ES1-expressing rods. Consistent with the mitochondrial enlargement, mitochondria-related genes involved in electron transport and oxidative phosphorylation were also significantly up-regulated (Fig. 4a, Table 1 and Supplementary Fig. S8). Key transcription factors required for mitochondrial biogenesis, such as estrogen-related receptor alpha (ERRα)^16^ and mitochondrial transcription factor (TFAM)^17^, were significantly up-regulated (Fig. 4b,c), suggesting that mitochondrial biogenesis was facilitated in the ES1-expressing rods. Consistent with the up-regulation of TFAM, which is also known to play an essential role to regulate mitochondrial DNA (mtDNA) content^17^, the ES1-expressing rods contained significantly higher amount of mtDNA than the wild-type rods (Fig. 4d). Expressions of fusion- and fission-related genes were unchanged in the ES1-expressing rods (Fig. 4b,c), suggesting fusion-independent mitochondrial enlargement.

As to the down-regulated genes, aminoacyl-tRNA ligase was the only significantly overrepresented GO term and no significantly enriched pathway was detected (Supplementary Fig. S8).

### ES1 expression led to high energy state in rods

**Figure 5:**
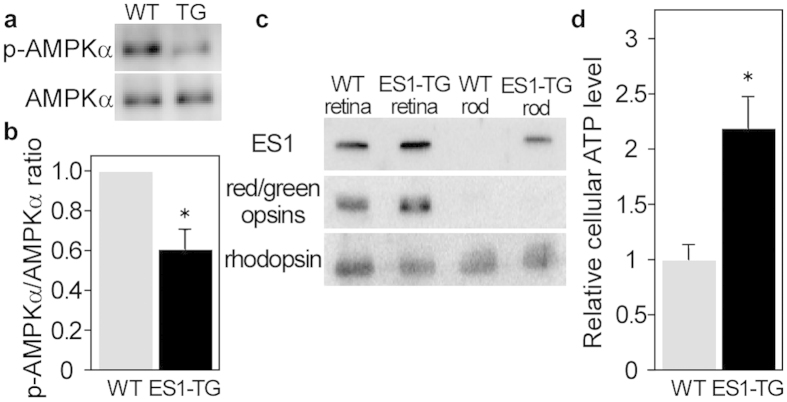
ES1-enhanced mitochondrial energy production in rods. (**a**) A representative result of immunoblottings with antibodies against AMPKα and phospho-AMPKα for estimation of phosphorylation levels of AMPKα in rods isolated from ES1-TG or WT siblings. (**b**) Relative phosphorylation levels of AMPKα in isolated rods assessed by the immunoblottings. Values are means ± S.E., *P = 0.008 in Student’s t-test, n = 7 for both WT and ES1-TG. (**c**) Immunoblottings of both the retinas and rods isolated from ES1-TG or WT siblings with antibodies against ES1, red/green opsins or rhodopsin. (**d**) Relative ATP levels in an isolated rod. Values are means ± S.E., *P = 0.002 in Student’s t-test, n = 8 for both ES1-TG and WT.

To investigate whether the mitochondria enlarged by ES1 possess high ATP production ability, we estimated the cellular energy state by measuring both phosphorylation levels of AMPKα and cellular ATP levels in rods purified either from the ES1-TG or the wild-type. AMPKα is a crucial sensor for cellular energy state^18^: in response to an elevated AMP/ATP ratio (low energy state), AMPKα is phosphorylated at Thr172 (in human AMPKα) and activated. The phosphorylation level of AMPKα in the ES1-expressing rods was suppressed by 60% of that in the wild-type rods (Fig. 5a,b), representing a high energy state in the ES1-expressing rods. Note that no contamination of cones was detected in the purified rod fractions (Fig. 5c). Consistently, cellular ATP levels in the ES1-TG rods were approximately twofold higher than that in the wild-type rods (Fig. 5d), indicating that the mitochondria enlarged by ES1 were capable of high ATP production. From these results, we can speculate that ES1 supports mitochondrial energy production in cones via mitochondrial enlargement.

## Discussion

The size of individual mitochondria has recently been known to be regulated by a dynamic equilibrium between fusion and fission. On the other hand, total mitochondrial mass is unrelated to the mitochondrial fusion or fission: neither overexpression of a mitochondrial fusion protein, mitofusin 1, nor knocking-out of a mitochondrial fission protein, dynamin related protein 1, increased total mitochondrial mass or cellular ATP levels^19^,^20^. In contrast, in the present study, ectopic ES1 expression increased the total mitochondrial mass (Fig. 3a,b) and the ATP production (Fig. 5) in rods without affecting the number of mitochondria (Fig. 3e), suggesting strongly that ES1-induced mitochondrial enlargement was achieved in a fusion-independent manner. Consistently, the RNA-seq data and the following validation by real-time PCR suggested that mitochondrial fusion was not increased in the ES1-expressing rods (Fig. 4b,c).

Since mega-mitochondria grow during development of cones with concomitant increase in total mitochondrial mass^10^, the ES1-induced enlargement is presumably a part of mitochondrial biogenesis, which has been known to be regulated by a transcriptional regulator, proliferator activated receptor gamma coactivator 1α (PGC-1α), and its downstream genes^21^. Overexpression of PGC-1α induced mitochondrial enlargement with concomitant increase in the total mitochondrial mass, mt DNA content and ATP production in cultured murine myoblasts^22^. These phenotypes agreed well with those observed in the ES1-expressing rods (Figs 3, 4d and 5), even though PGC-1α and its related gene, PGC-1β, were not up-regulated (Fig. 4b,c). Previous studies have shown that ES1 gene was up-regulated in PGC-1α-overexpressing murine myoblasts^23^ and down-regulated in PGC-1α knockout or depleted mice^24^,^25^, indicating that ES1 acts downstream of PGC-1α. These facts suggest that ES1 plays an important role in mitochondrial biogenesis in the downstream of PGC-1α.

The expression of ES1in rods up-regulated ERRα (Fig. 4c), which is a well-established downstream transcription factor of PGC-1α in the mitochondrial biogenesis^16^,^26^. Overexpression of ERRα increased total mitochondrial mass in myocytes^27^,^28^, and ERRα null mice showed decreased mitochondrial mass^29^. In the ES1-expressing rods, a number of nuclear-encoded mitochondrial genes were up-regulated (Fig. 4a, Table 1 and Supplementary Fig. S8) and a large portion of these genes (*ckmt2*, *coq4*, *cox6a1*, *gfm1*, *gfm2*, *glrx5*, *got2a*, *ndufa6*, *ndufs1*, *ndufs3*, *mrps30* and *uqurq*) have been predicted to be direct transcriptional targets of ERRα^26^,^30^,^31^,^32^. In addition, TFAM has also been known to be a downstream gene of ERRα^33^. Since TFAM is a major transcription factor for mtDNA-encoded genes, up-regulation of TFAM in the ES1-expressing rods (Fig. 4c) would enhance expression of the mtDNA-encoded genes. Collectively, our data strongly suggest that ES1 enhances expressions of both the nuclear and mtDNA-encoded mitochondrial genes to facilitate mitochondrial enlargement via up-regulation of ERRα.

Although the expression of ES1 is restricted in cones (Fig. 1a–d), ES1 paralogue (Unigene ID: Dr.82544), which possesses a putative MLS at the N-terminus, is expressed in diverse tissues^34^. Similarly, human (Unigene ID: Hs.413482) and mouse (Unigene ID: Mm.268691) orthologues of ES1 are also expressed in diverse tissues, especially in tissues with high metabolic demands such as heart, kidney and muscle^34^, and are also considered to be localized in mitochondria^35^. These facts imply that ES1 homologues are involved in mitochondrial biogenesis in diverse cell types.

Despite a large number of recent studies on mitochondrial biogenesis, molecular mechanisms underlying enlargement of individual mitochondria during the mitochondrial biogenesis have been largely unknown. To our knowledge, no mitochondrial proteins have been reported to promote the mitochondrial enlargement. Our present study led us to conclude that ES1 is the mitochondrial protein that was first found to promote the mitochondrial enlargement during the mitochondrial biogenesis in a fusion-independent manner in vertebrate photoreceptors.

## Methods

Detailed protocols for antibodies, subcellular fractionation, *in situ* hybridization, immunohistochemical studies, electron microscopic studies, gene expression analysis for RNA-seq and quantification of mtDNA content are provided in Supplementary Methods.

### Animals

Zebrafish of wild type Tübingen long fin (TL) line were subjected to all experiments. Adult zebrafish were reared in a continuous flow system (REI-SEA, IWAKI, Tokyo) at 28 °C under a 14 h light-10 h dark cycle, and larvae were reared in culture dishes at 28 °C under the same light-dark cycle. All experimental protocols were approved by Osaka University Graduate School of Frontier Biosciences (approval number, FBS 14-006). All experimental procedures involving animals and their care were carried out in accordance with the Osaka University Guidelines for Animal Experimentation and the National Institutes of Health Guide for the Care and Use of Laboratory Animals. In all experiments using TG or MO-injected zebrafish, control fish were treated in parallel with the same experimental procedures at every time points.

### RT-PCR

Total RNAs were extracted from adult zebrafish tissues, whole embryos and a single eye of embryos with TRI REAGENT (Sigma-Aldrich) according to the manufacture’s instruction. Ultra Pure glycogen (Invitrogen) was used as a carrier for isopropanol precipitation. The purified RNAs were reverse transcribed by SuperScript III reverse transcriptase (Invitrogen). In all RT-PCR assays, we performed nested PCR with two primer sets: 5′-GCACGAGGTCACTTTATCTCTC-3′ (forward primer) and 5′-AGAGGAGCCATGCTGGACAA-3′ (reverse primer) were used for the first round of the amplification, and 5′-AGTAAATCAGTCATGTTGGCATCTC-3′ (forward primer) and 5′-TGGAGCAAAGATCTGAAAACGG-3′ (reverse primer) were used for the second round.

### Antibodies

Anti-ES1, mAAT, rhodopsin, red/green opsins, cone-type arrestin-1 and -2 antibodies were prepared in our laboratory or raised commercially. Antigens used for production of each antibody were described in Supplementary Methods. The other antibodies used were commercially available; anti-alpha-tubulin (Sigma-Aldrich), anti-TOM20 (Santa Cruz Biotechnology, inc.), anti-AMPKα (Cell Signaling Technology) and anti-phospho-AMPKα antibodies (Cell Signaling Technology). Each antibody was used at dilutions as described in Supplementary Methods.

### MO-mediated ES1 knockdown

Two types of MOs against ES1 transcript were synthesized by Gene Tools LLC (Supplementary Fig. S3). The sequence of ES1-MO1 was 5′-CAGAGAAAACCTTCAACAGCGCAGA -3′, and ES1-MO2 was 5′-AGAGCCCGTGATGCCAACATGACTG-3′. Standard control MO (Gene Tools LLC) was used as a negative control. Approximately 0.5 nl of 1 mM MO solution (approximately 4.2 ng MO/embryo) was injected into each zebrafish embryo at 1–4 cell-stage.

### Immunoelectron microscopy

The ultrathin sections prepared as described in Supplementary Methods were immunostained as described elsewhere^36^ with minor modifications. Briefly, the sections were incubated in an activating buffer (20 mM Tris-HCl, 0.15 M NaCl, pH 9.0) at 95 °C for 1 h followed by incubation in a blocking solution containing 1% (w/v) bovine serum albumin in TBS (20 mM Tris-HCl, 0.15 M NaCl, pH 7.4) at room temperature for 30 min. The sections were then incubated with anti-TOM20 antibody (1:800 dilution) in the blocking solution at 4 °C overnight. After washing in the blocking solution, the sections were incubated with anti-mouse IgG-conjugated with colloidal-gold (10 nm diameter, British Biocell International, 1:60 dilution) in the blocking solution at room temperature for 30 min. After washing in TBS, the sections were fixed with 0.1 M phosphate buffer (pH 7.4) containing 1% (w/v) glutaraldehyde at room temperature for 10 min. Then the sections were washed in the phosphate buffer three times, in distilled water once, and then post-stained as described in Supplementary Methods.

### Generation of transgenic zebrafish

We used *Tol*2 tranposon system^37^,^38^ to generate transgenic zebrafish. *Tol*2-based expression vectors were constructed as shown in Supplementary Fig. S6. Approximately 1 nl of an injection solution (5 mM HEPES, pH 7.6, 150 mM KCl, 0.05% phenol red, 25 ng/μl plasmid DNA of each expression construct and 25 ng/μl *Tol*2 transposase mRNA) was injected into fertilized eggs. The embryos were reared at 27.5 °C and founder larvae were selected with the aid of fluorescence of EGFP in the eyes.

### RNA-seq

Retinas were isolated from the light-adapted ES1-TG and EGFP-TG zebrafish at 8 h after light onset. The retinas were chopped and incubated at room temperature for 15 min in Ringer’s solution with 10 Wünsch Units/ml liberase (Roche) and 20 U/ml hyaluronidase (Sigma-Aldrich) to dissociate into single cells. The cells were precipitated by centrifugation at 500 × g for 2 min and resuspended in Ringer’s solution. Rod cell bodies were sorted by EGFP signal intensity (over 10,000) and size (forward scatter value ranging between 50,000–100,000) using BD FACS Aria III flow cytometry (BD Bioscience), and immediately centrifuged at 2,000 × g for 30 sec to remove supernatant and frozen until mRNA preparation. Purification of mRNA was performed with Dynabeads^®^ mRNA DIRECT™ Micro Purification Kit (Life Technologies) according to the manufacturer’s protocol. Quantitative estimation and quality evaluation of the mRNA were performed using Agilent 2100 bioanalyzer with RNA Pico Chips (Agilent Technologies). The purified mRNA samples with RNA integrity number (>8.0) were sequenced by Ion PGM device with Ion total RNA-seq kit v2, Ion PGM Template OT2 kit and Ion PGM 200 Sequencing kit v2 (Life Technologies) according to the manufacturer’s protocol. The sequence dataset was submitted to the DDBJ Sequence Read Archive (DRA) under accession number DRA004229 with the following BioProject accession number PRJDB4416 and BioSample accession numbers SAMD00044052–SAMD00044057.

### Quantitative real-time PCR

Total RNAs were extracted from the EGFP- and ES1-TG purified rods (see “RNA-seq” in Methods) by using TRI REAGENT (Sigma-Aldrich) and treated with RQ1 RNase-Free DNase (Promega). Complementary DNAs synthesized as described above were used for Real-time PCR by Applied Biosystems 7900 HT Fast Real-Time PCR System (Life Technologies) with SYBR Green Realtime PCR Master Mix (TOYOBO) in two technical replicates. Specificities of amplified products in each experiment were validated by melting curve analyses. Expression levels were normalized to that of alpha-tubulin as the internal control. Primer set for ERRα was 5′-AGATGTGGCATCTGGCTACC-3′ (forward) and 5′-GCCTACTTTGAGGCACTTGG-3′ (reverse), and for the other genes were described previously; alpha-tubulin^39^, TFAM^40^, OPA1^41^, DRP1^41^ and PGC-1^41^.

### Estimation of cellular energy states of rods

Retinas were isolated from dark-adapted (more than 3 h) adult zebrafish at time points of 4–6 h after light onset and gently shaken in Ringer’s solution to isolate rods under dim red light. The rods were then purified by Percoll density gradient centrifugation^42^ at 10,000 × g for 10 min, and collected from a boundary between 45% and 60% (w/v) under dim red light. The collected rods were composed of outer segments and ellipsoids without cell bodies or synaptic termini. The rods were then precipitated by a serial centrifugations; 600 × g for 12 s followed by 3,000 × g for 4 s. The precipitate was suspended in Ringer’s solution and used to estimate both the phospho-AMPKα and the cellular ATP levels. ATP levels were measured with a luciferin-luciferase reaction assay method using an ATP assay kit for tissue (Toyo Ink) according to the manufacturer’s protocol. Signal intensity for luminescence was measured by using LAS2000 imaging system (Fuji Photo Film) with the aid of Image Gauge software (Fuji Photo Film).

## Additional Information

**How to cite this article**: Masuda, T. *et al.* ES1 is a mitochondrial enlarging factor contributing to form mega-mitochondria in zebrafish cones. *Sci. Rep.* **6**, 22360; doi: 10.1038/srep22360 (2016).

## Supplementary Material

Supplementary Information

## Figures and Tables

**Table 1:** Significantly enriched pathways with up-regulated genes in the ES1-expressing rods.

Pathway	Number of genes (O)	Expected number of genes (E)	Ratio of enrichment (O/E)	P value	Q value
protein synthesis (cytoplasmic ribosomal proteins)	8	2.36	3.40	0.0023	0.018
electron transport	7	2.04	3.43	0.0040	0.018
oxidative phosphorylation	5	1.27	3.95	0.0080	0.024

Adjustment of statistical significance levels for multiple testing was performed by Benjamini and Hochberg procedure, Q < 0.05. Minimum number of genes for a pathway was five.
