# Supplementary material for: ES1 is a mitochondrial enlarging factor contributing to form mega-mitochondria in zebrafish cones
Source: Sci Rep. 2016 Mar 1;6:22360. doi: 10.1038/srep22360 (PMC4772128; doi:10.1038/srep22360)
Supplement: Supplementary Information [file srep22360-s1.pdf]

# Supplementary Information

## ES1 is a mitochondrial enlarging factor contributing to form mega-mitochondria in zebrafish cones

Takamasa Masuda[1], Yasutaka Wada[1, 2, *], and Satoru Kawamura[1, 2,*]

[1]Graduate School of Frontier Biosciences, Graduate School of Science, Osaka University, Suita, Osaka 565-0871, Japan.

[2]Department of Biological Sciences, Graduate School of Science, Osaka University, Suita, Osaka 565-0871, Japan.

[*]Correspondence to: Yasutaka Wada
Graduate School of Frontier Biosciences, Osaka University,
Suita, Osaka 565-0871, Japan.
e-mail: ywada@fbs.osaka-u.ac.jp
TEL: +81- 6-6879-4610
FAX: +81- 6-6879-4614

and

Satoru Kawamura,
Graduate School of Frontier Biosciences, Osaka University,
Suita, Osaka 565-0871, Japan.
e-mail: kawamura@fbs.osaka-u.ac.jp
TEL: +81- 6-6879-4610
FAX: +81- 6-6879-4614

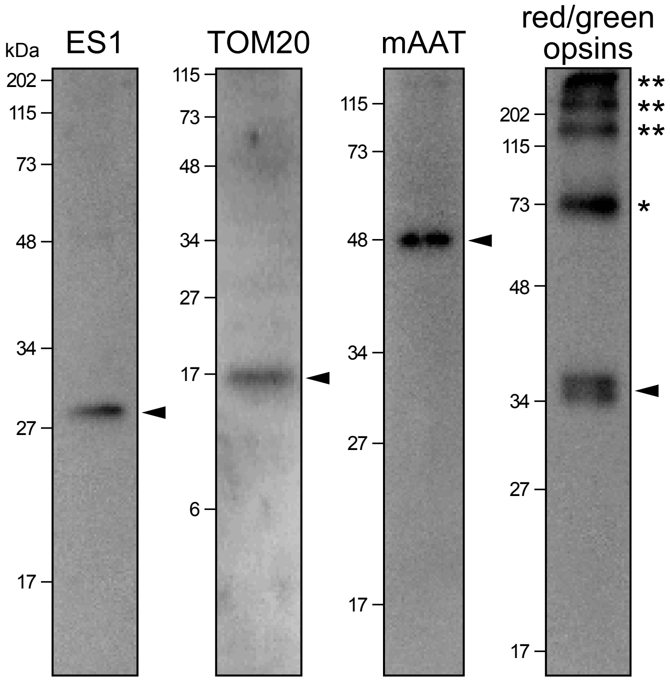


**Supplementary Figure S1. Immuno-specificities of antibodies used for immunohistochemical analyses.** Immunoblottings were performed with anti-ES1, TOM20, mAAT or red/green opsins antibodies on the lysate from the zebrafish eyes. Anti-ES1, TOM20 and mAAT antibodies showed only single bands (arrowheads), which correspond to the calculated molecular masses of each of the proteins. Anti-red/green opsins antibody showed multiple bands which correspond to monomers (arrowhead), dimers (single asterisk) or multimers (double asterisks) of red/green opsins as previously reported [1].

a

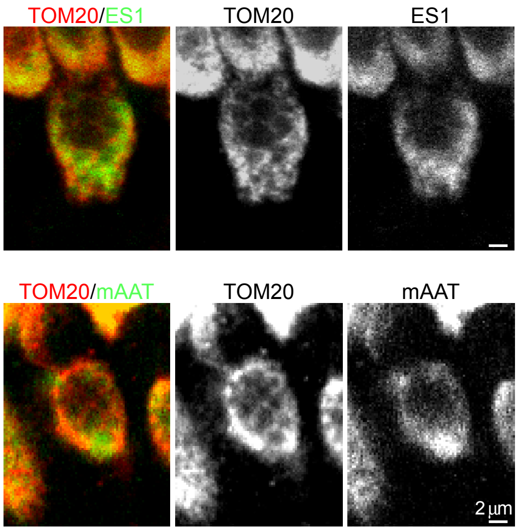


b

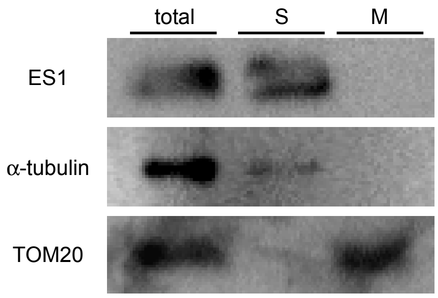


**Supplementary Figure S2. Subcellular localization of ES1 in zebrafish cones.** (**a**) Apparent heterogeneity of immunoreactivities in cone ellipsoid. High magnification views of double immunostainings of cone ellipsoids with TOM20/ES1 antibodies (upper panels) and TOM20/mAAT antibodies (lower panels). TOM20 antibody was used as a mitochondrial outer membrane marker to detect the outline of individual mitochondria. Both ES1- and mAAT-immunopositive signals were weaker at apical (upward direction) and central regions of the cone ellipsoids (right panels). In the same regions, TOM20 antibody depicted clear round patches >2 µm in diameter (middle panels), indicating the presence of mega-mitochondria. (**b**) Subcellular fractionation analysis for ES1. Immunoblotting analysis with anti-ES1 antibodies was performed using fractionated cells from retinas. Anti-alpha-tubulin and TOM20 antibodies were used as a marker for soluble fraction and membrane fraction, respectively. S: soluble fraction, M: membrane fraction.

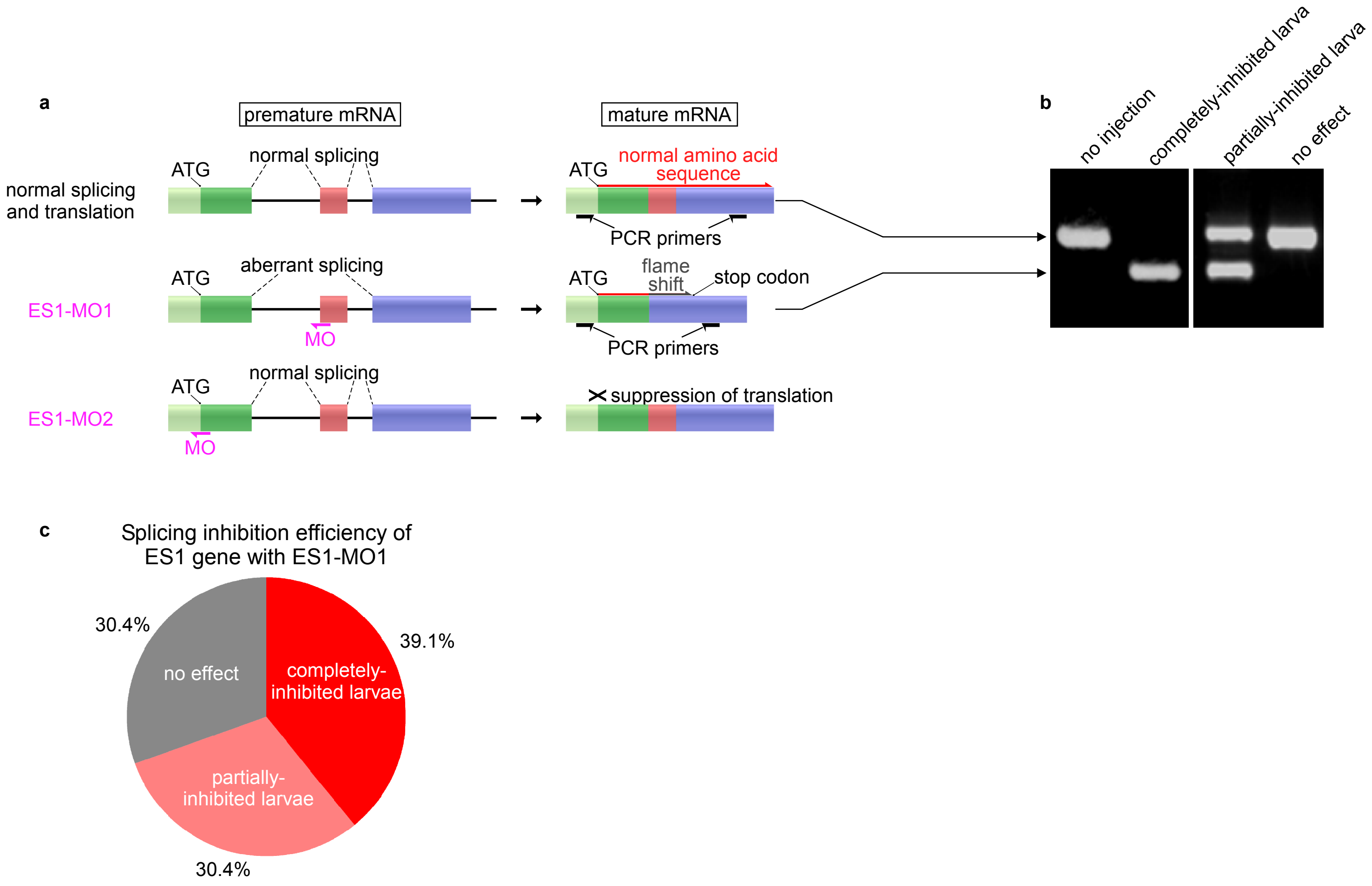


**Supplementary Figure S3. Two knock-down strategies for ES1 by using MOs.** (**a**) Schematic drawings of the exon-intron structure of the ES1 gene and expected effects of two MOs. Thick coloured bars and thin lines indicate exons and introns, respectively. ES1-MO1 was designed to hybridize to an intron-exon boundary, leading to an exon-skipping to form a frame-shifted mRNA coding only a short aberrant sequence (24 amino acids) following N-terminal mitochondrial localization signal (23 amino acids). ES1-MO2 was designed to hybridize in close proximity to the translation initiation site of ES1 mRNA and to suppress the translation. (**b**) Representative results of RT-PCR assay evaluating the effect of ES1-MO1. Template RNAs were extracted from larvae at 4 dpf stage. The upper band was derived from the normally-spliced mRNA and the lower band was derived from aberrantly-spliced mRNA. (**c**) Splicing inhibition efficiency of ES1 transcript with ES1-MO1. Splicing was completely inhibited (red) in 39%, partially inhibited (pink) in 30% and not inhibited (gray) in 30% of ES1-MO1-injected larvae. n=23. Dead or malformed larvae were eliminated from the calculation.

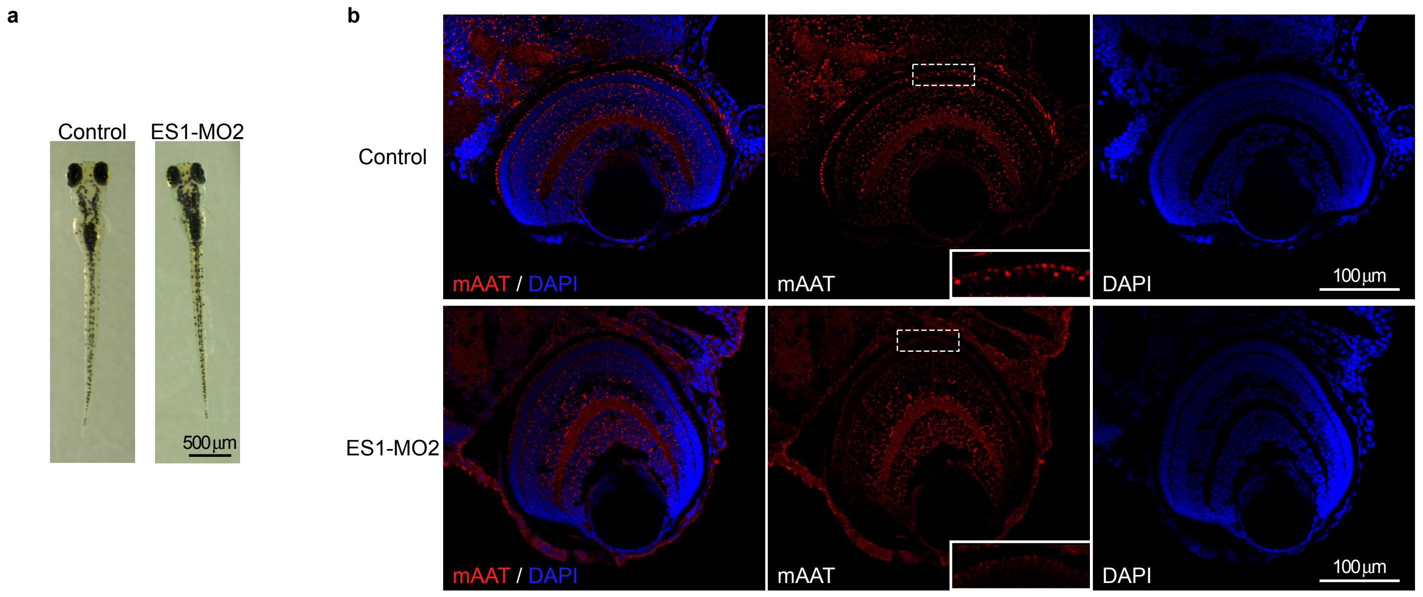


**Supplementary Figure S4. Morphology of MO-injected larvae.** (**a**) Representative whole body images of control MO- and ES1-MO2-injected 4 dpf larvae. (**b**) Immunohistochemistry of whole eye sections of control MO- and ES1-MO2-injected 4dpf larvae with mAAT antibody (red). Nuclei were stained with DAPI (blue). Immunopositive signals for mAAT were reduced only in cone ellipsoid layer (see dashed boxes and their magnified views shown in insets) of ES1-MO2-injected larvae compared to the control larvae.

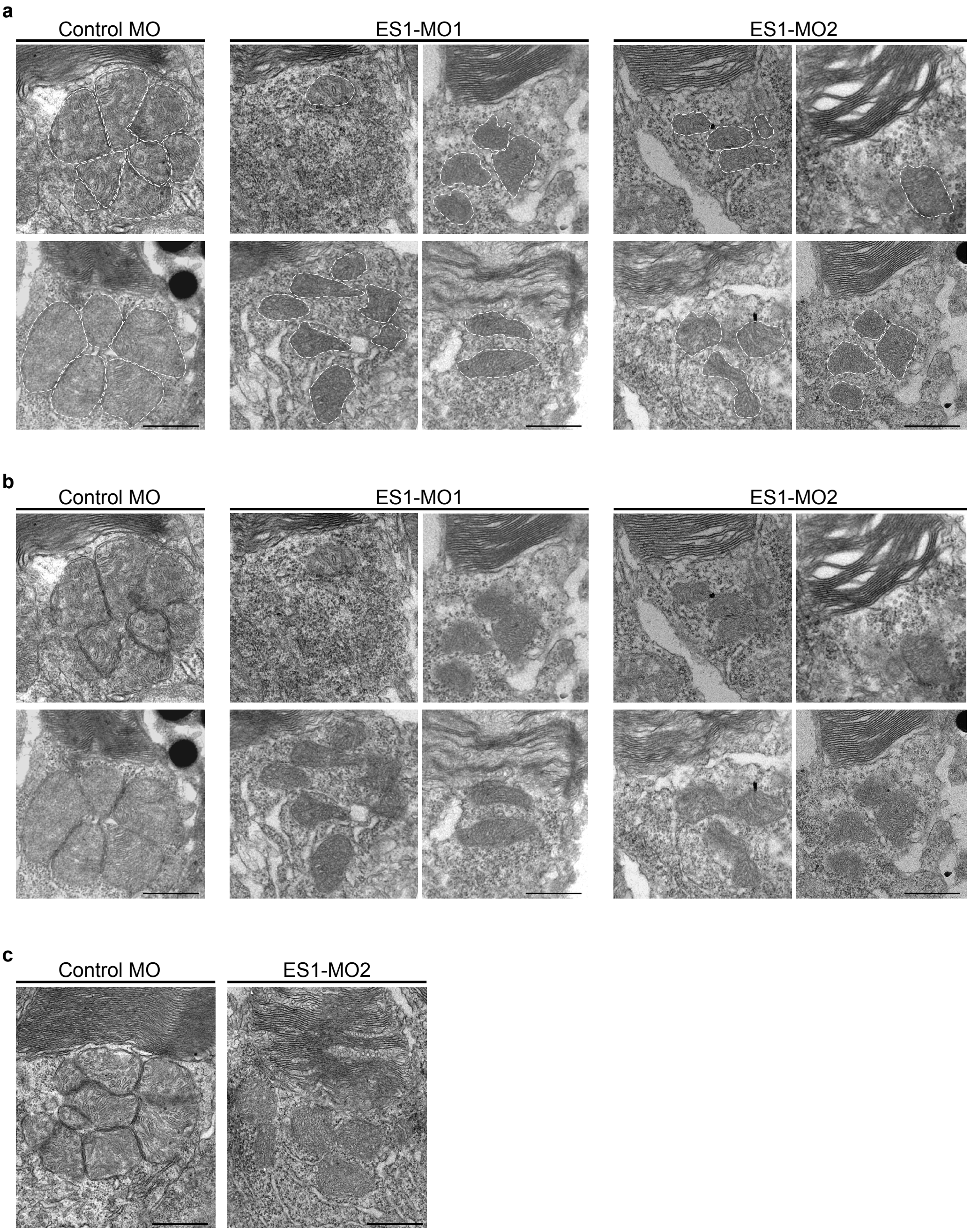


**Supplementary Figure S5. Morphology of cone mitochondria of MO-injected larvae.** (**a**) Representative electron microscopic images of cone mitochondria of control MO-injected (left panels), ES1-MO1-injected (center panels) or ES1-MO2-injected (right panels) larvae at 4 dpf. Dashed lines outline each mitochondrion. (**b**) Images of **a** without dashed lines. (**c**) Images of Fig. 2e without dashed lines. Scale bars, 1 μm.

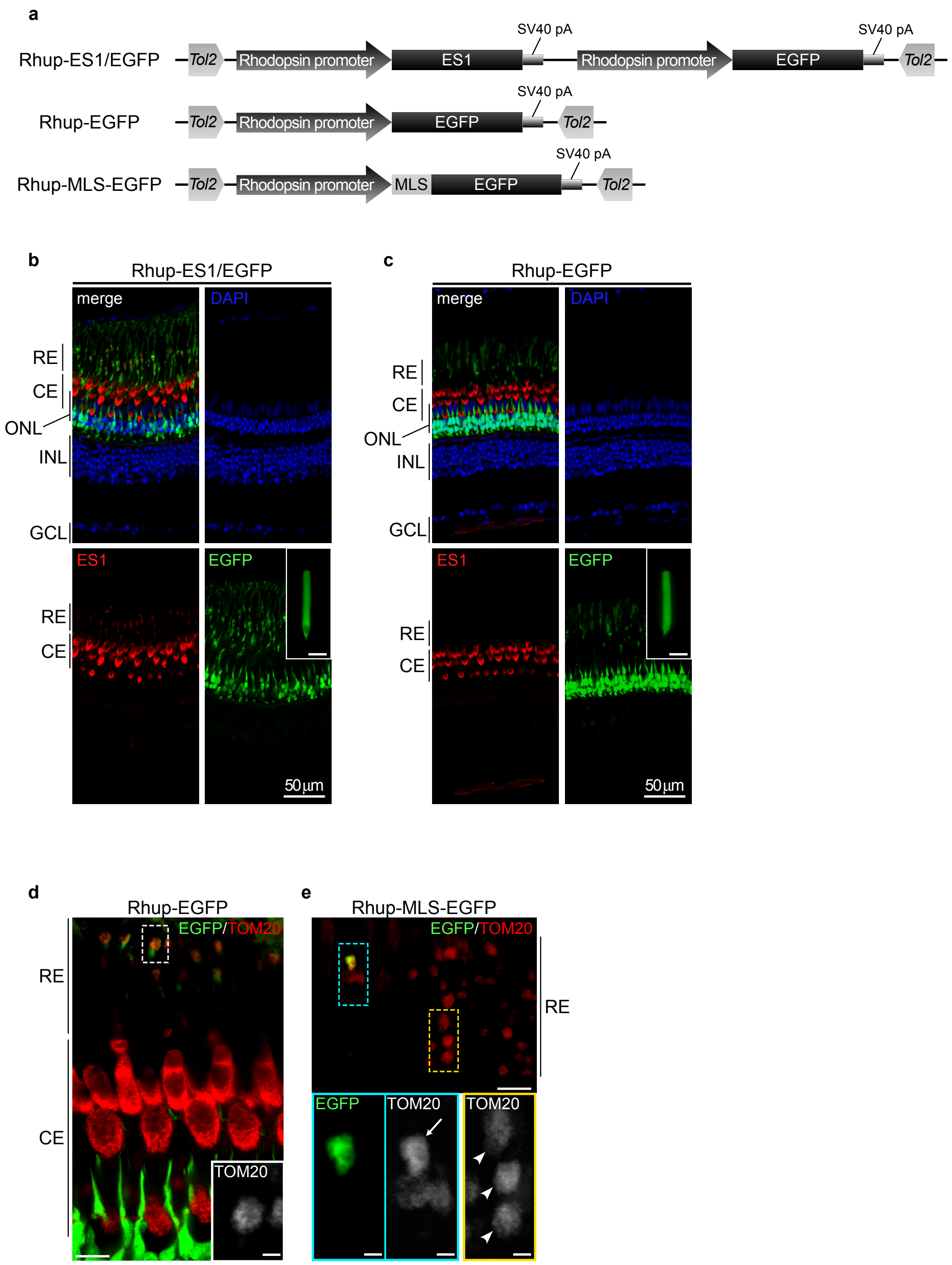


**Supplementary Figure S6. Ectopic expression of transgenes in zebrafish rods with the aid of *Tol2* transposon system.** (**a**) Schematic drawings of *Tol2*-based expression constructs. Coding sequences of ES1, EGFP and MLS-EGFP were driven by a sequence upstream 1,084 bp of rhodopsin gene [2]. Each construct was flanked by inverted repeats of *Tol2* transposon. Rhup-ES1/EGFP construct was used for the expression of ES1 and a reporter, EGFP, in rods. Rhup-EGFP and Rhup-MLS-EGFP constructs were used as controls. MLS-EGFP contains MLS of COX8 at the N-terminus [3]. (**b** and **c**) Immunohistochemistries of retinal sections of TG zebrafish harboring Rhup-ES1/EGFP (**b**, $F_0$ mosaic fish of the ES1-TG) or Rhup-EGFP (**c**, $F_1$ non-mosaic fish of the EGFP-TG) with ES1 antibody (red). EGFP signals are represented in green. Nuclei were stained with DAPI (blue). An isolated rod outer segment and ellipsoid from each retina is shown in each inset. Scale bars, 10 µm in the insets. (**d** and **e**) Immunohistochemistries of retinal sections of EGFP-TG (**d**) or TG harboring Rhup-MLS-EGFP (MLS-EGFP-TG; $F_0$ generation; mosaic, **e**) with TOM20 antibody (red). The inset in **d** and the lower panels in **e** are magnified views of areas surrounded by dashed lines in **d** and in the top panel in **e**, respectively. In panel **e**, an ellipsoid of MLS-EGFP-TG rod and ellipsoids of wild-type rods are indicated by an arrow and arrow heads, respectively. Scale bars, 10 µm in the main panel of **d** and the upper panel of **e**, and 2 µm in the inset of **d** and the lower panels of **e**. RE: rod ellipsoid, CE: cone ellipsoid, ONL: outer nuclear layer, INL: inner nuclear layar, GCL: ganglion cell layer.

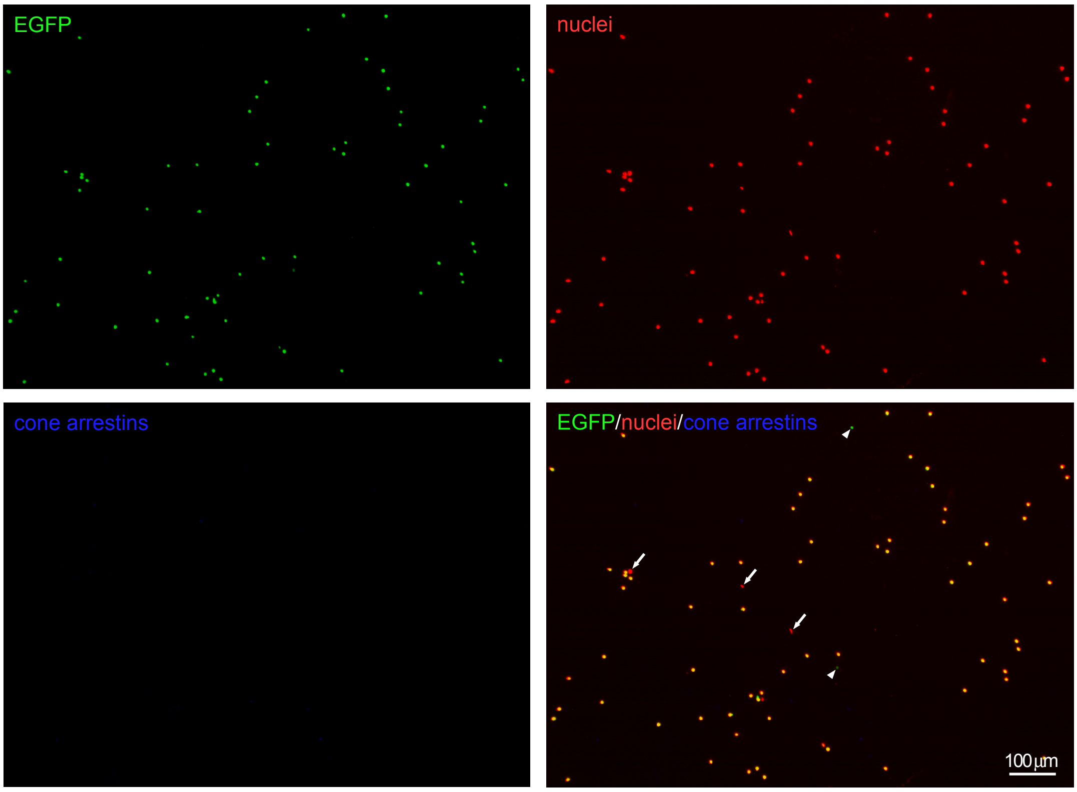


**Supplementary Figure S7. Purity of FACS-purified rods**. Representative microscopic images of a purified rod fraction from ES1-TG. Rod cell bodies were identified by signals for both EGFP (green) and nuclei (red, stained with DAPI). Approximately 97% of nuclei-containing dots were EGFP-positive. Few dots showed signal only for either EGFP (arrow heads) or nuclei (arrows), which were derived from rod outer segment or other cells, respectively. Possible contamination of cones was also investigated by immunostaining with antibodies against cone arrestins. Population of cone arrestins-immunopositive cells was only 0.28%.

**a** Up-regulated genes

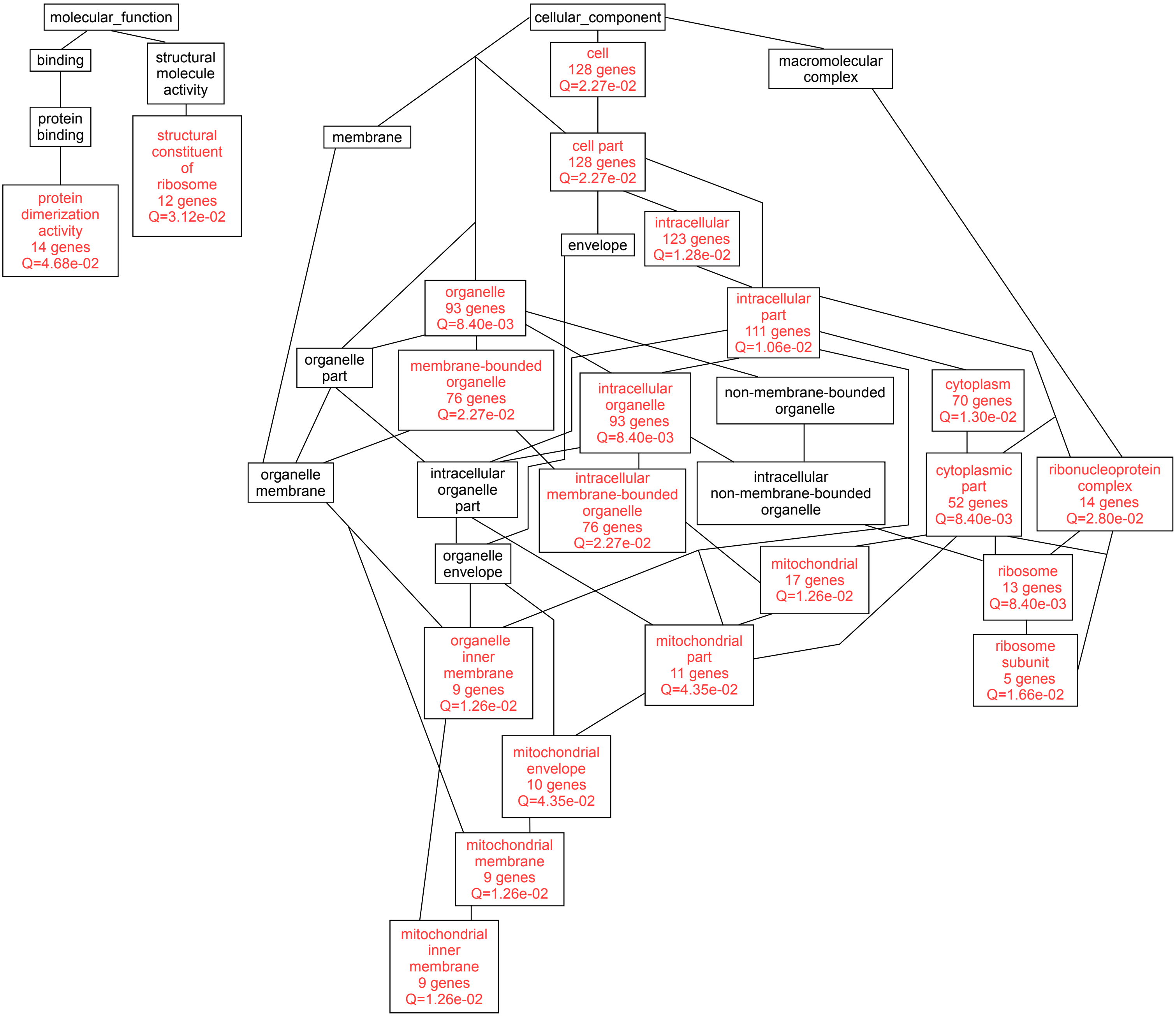


**b** Down-regulated genes

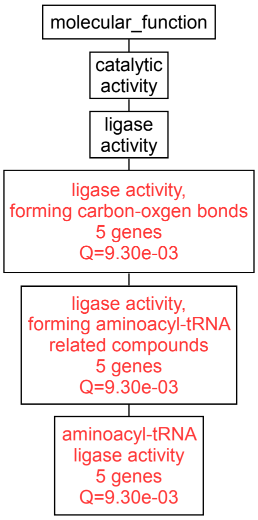


**Supplementary Figure S8. Directed acyclic graph of the enriched GO categories of differentially expressed genes between the ES1- and EGFP-expressing rods.** GO analyses based on the RNA-seq data were performed on more than 1.5 fold up-regulated (**a**) or down-regulated (**b**) genes. In multiple test adjustment performed by Benjamini and Hochberg procedure, significantly enriched (Q >0.05) and not enriched GO categories are represented in red and black, respectively. Minimum number of genes for a GO category was five.

# Supplementary Table S1

Up-regulated genes in the ES1-expressing rods. Genes were sorted in descending order according to the value of fold change.

| Genbank accession number | Gene symbol | Gene description | fold change | P value | Q value |
|---|---|---|---|---|---|
| NM_131039 | es1 | es1 protein | 476.41 | 5.0E-5 | 0.005 |
| NM_001006039 | *samsn1b* | SAM domain, SH3 domain and nuclear localisation signals, 1b | 49.90 | 5.0E-5 | 0.005 |
| NM_131256 | *nr6a1a* | nuclear receptor subfamily 6, group A, member 1a | 8.60 | 5.0E-5 | 0.005 |
| NM_001113659 | zgc:198419 | zgc:198419 | 6.80 | 0.002 | 0.084 |
| NM_001085494 | *hoxa13a* | homeo box A13a | 5.19 | 5.0E-5 | 0.005 |
| NM_131249 | *evx1* | even-skipped homeobox 1 | 4.35 | 5.0E-5 | 0.005 |
| NM_001244736 | *cnbpa* | CCHC-type zinc finger, nucleic acid binding protein a | 4.05 | 2.5E-4 | 0.017 |
| NM_001109854 | zgc:173594 | zgc:173594 | 3.94 | 5.0E-5 | 0.005 |
| NM_001077315 | zgc:153911 | zgc:153911 | 3.80 | 5.0E-5 | 0.005 |
| NM_001002190 | *rgcc* | regulator of cell cycle | 3.80 | 5.0E-5 | 0.005 |
| NR_036645 | *rpph1* | ribonuclease P RNA component H1 | 3.77 | 0.010 | 0.206 |
| NM_001037573 | *actn2* | actinin, alpha 2 | 3.20 | 5.0E-5 | 0.005 |
| NM_200303 | zgc:56676 | zgc:56676 | 3.16 | 0.031 | 0.332 |
| NM_001020476 | *dnmt3b* | DNA (cytosine-5-)-methyltransferase 3 beta | 3.00 | 5.0E-5 | 0.005 |
| NM_131295 | *cpla2* | cytosolic phospholipase a2 | 2.90 | 5.0E-5 | 0.005 |
| NM_001162502 | *ncapd2* | non-SMC condensin I complex, subunit D2 | 2.90 | 5.0E-4 | 0.028 |
| NM_001098760 | *mul1b* | mitochondrial E3 ubiquitin ligase 1b | 2.88 | 0.006 | 0.146 |
| NM_001114720 | *coq4* | coenzyme Q4 homolog (S. cerevisiae) | 2.72 | 0.019 | 0.271 |
| NM_213180 | zgc:77112 | zgc:77112 | 2.69 | 5.0E-5 | 0.005 |
| NM_001126421 | LOC565395 | novel protein similar to DIRAS family, GTP-binding RAS-like 1 (diras1) | 2.62 | 0.008 | 0.182 |
| NM_131368 | *smad5* | MAD homolog 5 (Drosophila) | 2.60 | 5.0E-5 | 0.005 |

| Genbank accession number | Gene symbol | Gene description | fold change | P value | Q value |
|---|---|---|---|---|---|
| NM_001003533 | *bag3* | BCL2-associated athanogene 3 | 2.60 | 5.0E-5 | 0.005 |
| NM_001076749 | zgc:154164 | zgc:154164 | 2.56 | 2.0E-4 | 0.015 |
| NM_130936 | *klfd* | Kruppel-like factor d | 2.53 | 5.0E-5 | 0.005 |
| NM_001128780 | zgc:194423 | zgc:194423 | 2.52 | 0.007 | 0.171 |
| NM_200172 | *mycb* | myelocytomatosis oncogene b | 2.52 | 0.004 | 0.125 |
| NM_001039144 | *slc8a1b* | solute carrier family 8 (sodium/calcium exchanger), member 1b | 2.42 | 5.0E-5 | 0.005 |
| NM_199580 | *hmmr* | hyaluronan mediated motility receptor | 2.41 | 1.5E-4 | 0.012 |
| NM_001002075 | *ccne2* | cyclin E2 | 2.40 | 5.0E-5 | 0.005 |
| NM_001045854 | *pex11b* | peroxisomal biogenesis factor 11 beta | 2.40 | 0.027 | 0.312 |
| NM_001122611 | *magi2* | membrane associated guanylate kinase, WW and PDZ domain containing 2 | 2.36 | 5.0E-5 | 0.005 |
| NM_001077545 | *gfm2* | G elongation factor, mitochondrial 2 | 2.33 | 7.5E-4 | 0.039 |
| NM_001008644 | *bmp2k* | BMP2 inducible kinase | 2.31 | 5.0E-5 | 0.005 |
| NM_199850 | *c20orf24* | c20orf24 homolog (H. sapiens) | 2.29 | 3.0E-4 | 0.019 |
| NM_200167 | *cpne3* | copine III | 2.25 | 2.0E-4 | 0.015 |
| NM_001014343 | *sh2d5* | SH2 domain containing 5 | 2.17 | 0.002 | 0.067 |
| NM_131237 | *mxi1* | max interacting protein | 2.16 | 5.0E-5 | 0.005 |
| NM_200701 | *wdr21* | WD repeat domain 21 | 2.13 | 0.032 | 0.336 |
| NM_001110833 | *ccdc94* | coiled-coil domain containing 94 | 2.12 | 0.018 | 0.267 |
| NM_001201561 | LOC555548 | cardiac phospholamban-like | 2.12 | 0.007 | 0.163 |
| NM_199987 | *jun* | jun proto-oncogene | 2.10 | 5.0E-5 | 0.005 |
| NM_200802 | *clu* | clusterin | 2.08 | 5.0E-5 | 0.005 |
| NM_001020735 | *pcyt1ab* | phosphate cytidylyltransferase 1, choline, alpha b | 2.06 | 4.5E-4 | 0.026 |
| NM_001007340 | *ppm1db* | protein phosphatase 1D magnesium-dependent, delta isoform b | 2.06 | 0.009 | 0.190 |

| Genbank accession number | Gene symbol | Gene description | fold change | P value | Q value |
|---|---|---|---|---|---|
| NM_001014330 | *mtmr1b* | myotubularin related protein 1b | 2.05 | 7.5E-4 | 0.039 |
| NM_001002743 | *tk2* | thymidine kinase 2, mitochondrial | 2.01 | 0.011 | 0.216 |
| NM_001122705 | *abl2* | v-abl Abelson murine leukemia viral oncogene homolog 2 | 2.01 | 0.010 | 0.208 |
| NM_001001846 | *pura* | purine-rich element binding protein A | 2.00 | 0.003 | 0.104 |
| NM_001077605 | zgc:153381 | zgc:153381 | 1.99 | 0.013 | 0.233 |
| NM_001020551 | *hmg20b* | high-mobility group 20B | 1.99 | 0.020 | 0.278 |
| NM_200117 | *hist2h2l* | histone 2, H2, like | 1.98 | 5.0E-5 | 0.005 |
| NM_001102675 | *pard6b* | par-6 partitioning defective 6 homolog beta (C. elegans) | 1.98 | 0.048 | 0.385 |
| NM_001017678 | *ppie* | peptidylprolyl isomerase E (cyclophilin E) | 1.97 | 0.014 | 0.244 |
| NM_001113610 | si:ch211-163l21.7 | si:ch211-163l21.7 | 1.95 | 4.0E-4 | 0.024 |
| NM_001005587 | *crata* | carnitine O-acetyltransferase a | 1.95 | 0.008 | 0.187 |
| NM_001017840 | *rasl11a* | RAS-like, family 11, member A | 1.95 | 0.003 | 0.107 |
| NM_001130625 | zgc:194737 | zgc:194737 | 1.95 | 0.005 | 0.131 |
| NM_001077389 | tfam | transcription factor A, mitochondrial | 1.93 | 0.029 | 0.320 |
| NM_212612 | *ddx5* | DEAD (Asp-Glu-Ala-Asp) box polypeptide 5 | 1.91 | 5.0E-5 | 0.005 |
| NM_200102 | *pdcd8* | programmed cell death 8 (apoptosis-inducing factor) | 1.91 | 1.5E-4 | 0.012 |
| NM_212750 | *junbb* | jun B proto-oncogene b | 1.90 | 1.0E-4 | 0.008 |
| NM_001109835 | zgc:171937 | zgc:171937 | 1.88 | 0.034 | 0.344 |
| NM_199636 | *rpl10a* | ribosomal protein L10a | 1.87 | 2.5E-4 | 0.017 |
| NM_200233 | *hif1ab* | hypoxia-inducible factor 1, alpha subunit (basic helix-loop-helix transcription factor) b | 1.86 | 5.0E-5 | 0.005 |
| NM_001007765 | *ndufs1* | NADH dehydrogenase (ubiquinone) Fe-S protein 1 | 1.86 | 2.0E-4 | 0.015 |
| NM_199629 | *nfkbiab* | nuclear factor of kappa light polypeptide gene enhancer in B-cells inhibitor, alpha b | 1.84 | 1.0E-4 | 0.008 |
| NM_001044985 | si:dkey-72l14.3 | si:dkey-72l14.3 | 1.84 | 0.002 | 0.065 |

| Genbank accession number | Gene symbol | Gene description | fold change | P value | Q value |
|---|---|---|---|---|---|
| NM_213256 | *ttyh2l* | tweety homolog 2, like | 1.83 | 0.020 | 0.273 |
| NM_200789 | zgc:73324 | zgc:73324 | 1.83 | 0.017 | 0.262 |
| NM_001110286 | *prkaa1* | protein kinase, AMP-activated, alpha 1 catalytic subunit | 1.83 | 0.004 | 0.118 |
| NM_001004542 | zgc:91887 | zgc:91887 | 1.82 | 0.008 | 0.187 |
| NM_001098389 | *hexim1* | hexamethylene bis-acetamide inducible 1 | 1.82 | 0.022 | 0.283 |
| NM_200407 | *ergic2* | ERGIC and golgi 2 | 1.82 | 0.044 | 0.379 |
| NM_131073 | *jak1* | Janus kinase 1 | 1.80 | 0.005 | 0.134 |
| NM_199776 | *ppp2r2d* | protein phosphatase 2, regulatory subunit B, delta isoform | 1.80 | 0.005 | 0.137 |
| NM_001002304 | *twf1b* | twinfilin, actin-binding protein, homolog 1b | 1.79 | 0.018 | 0.267 |
| NM_001020543 | zgc:109982 | zgc:109982 | 1.79 | 0.011 | 0.210 |
| NM_001083123 | *ar* | androgen receptor | 1.79 | 0.026 | 0.309 |
| NM_200845 | *rps17* | ribosomal protein S17 | 1.78 | 0.041 | 0.370 |
| NM_213021 | *glrx5* | glutaredoxin 5 homolog (S. cerevisiae) | 1.78 | 0.035 | 0.349 |
| NM_153662 | *st8sia2* | ST8 alpha-N-acetyl-neuraminide alpha-2,8-sialyltransferase 2 | 1.78 | 7.5E-4 | 0.039 |
| NM_213408 | *slc25a22* | solute carrier family 25 (mitochondrial carrier: glutamate), member 22 | 1.78 | 0.023 | 0.293 |
| NM_205553 | zgc:55936 | zgc:55936 | 1.78 | 0.023 | 0.292 |
| NM_001017755 | *ndufs3* | NADH dehydrogenase (ubiquinone) Fe-S protein 3, (NADH-coenzyme Q reductase) | 1.76 | 0.009 | 0.191 |
| NM_001045015 | *ap3d1* | adaptor-related protein complex 3, delta 1 subunit | 1.76 | 0.001 | 0.055 |
| NM_201511 | *cyp2p10* | cytochrome P450, family 2, subfamily P, polypeptide 10 | 1.75 | 0.047 | 0.384 |
| NM_001045109 | *furinb* | furin (paired basic amino acid cleaving enzyme) b | 1.75 | 0.032 | 0.336 |
| NM_199272 | *esrp2* | epithelial splicing regulatory protein 2 | 1.75 | 2.0E-4 | 0.015 |
| NM_001029956 | *u2af2a* | U2 small nuclear RNA auxiliary factor 2a | 1.74 | 0.005 | 0.145 |
| NM_001089480 | zgc:162310 | zgc:162310 | 1.73 | 0.026 | 0.308 |

| Genbank accession number | Gene symbol | Gene description | fold change | P value | Q value |
|---|---|---|---|---|---|
| NM_001077257 | *foxo1a* | forkhead box O1 a | 1.73 | 0.020 | 0.276 |
| NM_001130603 | *tmtc2* | transmembrane and tetratricopeptide repeat containing 2 | 1.73 | 0.002 | 0.070 |
| NM_001082839 | *wdr85* | WD repeat domain 85 | 1.72 | 0.002 | 0.067 |
| NM_199992 | *sema6a* | sema domain, transmembrane domain (TM), and cytoplasmic domain, (semaphorin) 6A | 1.72 | 0.019 | 0.271 |
| NM_001177744 | *aqp9b* | aquaporin 9b | 1.70 | 0.005 | 0.131 |
| NM_001002512 | zgc:92818 | zgc:92818 | 1.70 | 0.008 | 0.187 |
| NM_199674 | *stub1* | STIP1 homology and U-Box containing protein 1 | 1.69 | 0.031 | 0.332 |
| NM_001103125 | *fam46a* | family with sequence similarity 46, member A | 1.69 | 0.011 | 0.211 |
| NM_001033091 | si:ch211-237l4.6 | si:ch211-237l4.6 | 1.69 | 0.003 | 0.109 |
| NM_001020663 | *bach1* | BTB and CNC homology 1, basic leucine zipper transcription factor 1 | 1.68 | 0.019 | 0.269 |
| NM_200179 | *cdc14b* | CDC14 cell division cycle 14 homolog B | 1.68 | 0.003 | 0.096 |
| NM_001167826 | zgc:73329 | zgc:73329 | 1.68 | 0.028 | 0.314 |
| NM_200989 | *pacsin3* | protein kinase C and casein kinase substrate in neurons 3 | 1.68 | 0.008 | 0.181 |
| NM_001002722 | *ttc33* | tetratricopeptide repeat domain 33 | 1.67 | 0.018 | 0.267 |
| NM_001003493 | *suds3* | suppressor of defective silencing 3 homolog (SDS3, S. cerevisiae) | 1.67 | 0.035 | 0.349 |
| NM_001007778 | *gpsm1b* | G-protein signaling modulator 1b | 1.66 | 0.037 | 0.353 |
| NM_001003479 | *rasgef1bb* | RasGEF domain family, member 1Bb | 1.66 | 0.020 | 0.278 |
| NM_201153 | *rps3* | ribosomal protein S3 | 1.66 | 0.004 | 0.128 |
| NM_199777 | *sec23b* | Sec23 homolog B (S. cerevisiae) | 1.66 | 0.017 | 0.265 |
| NM_130968 | *odz3* | odd Oz/ten-m homolog 3 | 1.66 | 0.007 | 0.165 |
| NM_213415 | *zbtb2b* | zinc finger and BTB domain containing 2b | 1.66 | 0.021 | 0.281 |
| NM_201315 | *abcf2a* | ATP-binding cassette, sub-family F (GCN20), member 2a | 1.65 | 0.005 | 0.137 |
| NM_200724 | *guk1b* | guanylate kinase 1b | 1.65 | 0.002 | 0.075 |

| Genbank accession number | Gene symbol | Gene description | fold change | P value | Q value |
|---|---|---|---|---|---|
| NM_198363 | *tob1a* | transducer of ERBB2, 1a | 1.64 | 0.003 | 0.101 |
| NM_001080036 | *tp53inp1* | tumor protein p53 inducible nuclear protein 1 | 1.64 | 0.014 | 0.240 |
| NM_001256204 | *elac2* | elaC homolog 2 (E. coli) | 1.64 | 0.032 | 0.334 |
| NM_001030234 | zgc:113372 | zgc:113372 | 1.64 | 0.045 | 0.381 |
| NM_001045485 | *pi4k2b* | phosphatidylinositol 4-kinase type 2 beta | 1.64 | 0.005 | 0.131 |
| NM_212645 | *hnrnpa0a* | heterogeneous nuclear ribonucleoprotein A0a | 1.63 | 0.003 | 0.101 |
| NM_200949 | *zfand5b* | zinc finger, AN1-type domain 5b | 1.63 | 0.010 | 0.199 |
| NM_199215 | *ca2* | carbonic anhydrase II | 1.63 | 0.002 | 0.066 |
| NM_001002355 | zgc:92139 | zgc:92139 | 1.63 | 0.008 | 0.182 |
| NM_001077379 | *sec23ip* | SEC23 interacting protein | 1.63 | 0.021 | 0.280 |
| NM_001126454 | *fam222a* | family with sequence similarity 222, member A | 1.62 | 0.041 | 0.370 |
| NM_001002708 | *pde6d* | phosphodiesterase 6D, cGMP-specific, rod, delta | 1.62 | 0.002 | 0.072 |
| NM_200276 | *ncor1* | nuclear receptor co-repressor 1 | 1.62 | 0.003 | 0.088 |
| NM_001006016 | *atl3* | atlastin 3 | 1.62 | 0.046 | 0.382 |
| NM_001110123 | *oscp1a* | organic solute carrier partner 1a | 1.62 | 0.002 | 0.066 |
| NM_001082998 | *fosl2* | fos-like antigen 2 | 1.62 | 0.012 | 0.222 |
| NM_001044859 | si:ch211-15d5.5 | si:ch211-15d5.5 | 1.62 | 0.029 | 0.318 |
| NM_001044848 | *arhgef1b* | Rho guanine nucleotide exchange factor (GEF) 1b | 1.61 | 0.020 | 0.273 |
| NM_212963 | *dip2ba* | DIP2 disco-interacting protein 2 homolog Ba (Drosophila) | 1.61 | 0.007 | 0.163 |
| NM_001080651 | *creb3l2* | cAMP responsive element binding protein 3-like 2 | 1.61 | 0.007 | 0.163 |
| NM_131081 | *cdh2* | cadherin 2, neuronal | 1.61 | 0.013 | 0.232 |
| NM_213198 | *orc5* | origin recognition complex, subunit 5 | 1.61 | 0.002 | 0.079 |
| NM_001077768 | *zranb1b* | zinc finger, RAN-binding domain containing 1b | 1.61 | 0.012 | 0.228 |

| Genbank accession number | Gene symbol | Gene description | fold change | P value | Q value |
|---|---|---|---|---|---|
| NM_001128534 | *arvcfb* | armadillo repeat gene deleted in velocardiofacial syndrome b | 1.60 | 0.004 | 0.116 |
| NM_213497 | *crip2* | cysteine-rich protein 2 | 1.60 | 0.011 | 0.216 |
| NM_212684 | *atxn2l* | ataxin 2-like | 1.60 | 0.020 | 0.276 |
| NM_001130663 | si:ch211-160i2.3 | si:ch211-160i2.3 | 1.59 | 0.048 | 0.385 |
| NM_199830 | *klhl18* | kelch-like 18 (Drosophila) | 1.58 | 0.015 | 0.248 |
| NM_001002495 | *uqcrq* | ubiquinol-cytochrome c reductase, complex III subunit VII | 1.58 | 0.043 | 0.375 |
| NM_001017800 | zgc:110331 | zgc:110331 | 1.58 | 0.003 | 0.102 |
| NM_200813 | *ca7* | carbonic anhydrase VII | 1.58 | 0.028 | 0.314 |
| NM_001128259 | *uckl1a* | uridine-cytidine kinase 1-like 1a | 1.58 | 0.004 | 0.124 |
| NM_199480 | *lnpa* | limb and neural patterns a | 1.58 | 0.019 | 0.269 |
| NM_001165918 | wu:fb15g10 | wu:fb15g10 | 1.58 | 0.023 | 0.293 |
| NM_001083075 | *znf865* | zinc finger protein 865 | 1.57 | 0.012 | 0.219 |
| NM_199541 | *id2b* | inhibitor of DNA binding 2, dominant negative helix-loop-helix protein, b | 1.57 | 0.019 | 0.269 |
| NM_001044786 | *shoc2* | soc-2 suppressor of clear homolog (C. elegans) | 1.57 | 0.018 | 0.267 |
| NM_001170739 | *mga* | MAX gene associated | 1.57 | 0.005 | 0.139 |
| NM_212974 | *tob1b* | transducer of ERBB2, 1b | 1.56 | 0.003 | 0.091 |
| NM_212955 | esrra | estrogen-related receptor alpha | 1.56 | 0.041 | 0.370 |
| NM_001034976 | *prkacbb* | protein kinase, cAMP-dependent, catalytic, beta b | 1.56 | 0.032 | 0.336 |
| NM_001044918 | *znf704* | zinc finger protein 704 | 1.56 | 0.026 | 0.306 |
| NM_001144784 | *inaa* | internexin neuronal intermediate filament protein, alpha a | 1.55 | 0.003 | 0.109 |
| NM_201470 | *ip6k2* | inositol hexaphosphate kinase 2 | 1.55 | 0.015 | 0.251 |
| NM_194380 | *dusp6* | dual specificity phosphatase 6 | 1.55 | 0.004 | 0.112 |
| NM_001127515 | si:dkey-110k5.6 | si:dkey-110k5.6 | 1.55 | 0.018 | 0.267 |

| Genbank accession number | Gene symbol | Gene description | fold change | P value | Q value |
|---|---|---|---|---|---|
| NM_131900 | *cacna1c* | calcium channel, voltage-dependent, L type, alpha 1C subunit | 1.55 | 0.009 | 0.198 |
| NM_201149 | *cdc14aa* | CDC14 cell division cycle 14 homolog A, a | 1.54 | 0.010 | 0.206 |
| NM_200064 | *bcat1* | branched chain aminotransferase 1, cytosolic | 1.54 | 0.012 | 0.222 |
| NM_001012378 | *dlg2* | discs, large (Drosophila) homolog 2 | 1.54 | 0.027 | 0.312 |
| NM_213149 | *fkbp5* | FK506 binding protein 5 | 1.54 | 0.037 | 0.353 |
| NM_212759 | *cyfip1* | cytoplasmic FMR1 interacting protein 1 | 1.53 | 0.009 | 0.198 |
| NM_001159971 | *spty2d1* | SPT2, Suppressor of Ty, domain containing 1 (S. cerevisiae) | 1.53 | 0.019 | 0.269 |
| NM_001098177 | *myh9a* | myosin, heavy polypeptide 9a, non-muscle | 1.53 | 0.005 | 0.144 |
| NM_001110279 | *mll* | myeloid/lymphoid or mixed-lineage leukemia (trithorax homolog, Drosophila) | 1.53 | 0.011 | 0.210 |
| NM_001007330 | *cbl* | Cas-Br-M (murine) ecotropic retroviral transforming sequence | 1.53 | 0.042 | 0.374 |
| NM_194399 | *cflar* | CASP8 and FADD-like apoptosis regulator | 1.52 | 0.028 | 0.318 |
| NM_001145089 | *camk2n1a* | calcium/calmodulin-dependent protein kinase II inhibitor 1a | 1.52 | 0.016 | 0.256 |
| NM_001030136 | *daam1b* | dishevelled associated activator of morphogenesis 1b | 1.52 | 0.016 | 0.261 |
| NM_001001940 | *copb2* | coatomer protein complex, subunit beta 2 | 1.52 | 0.020 | 0.276 |
| NM_212732 | *mdm4* | transformed 3T3 cell double minute 4 homolog (mouse) | 1.51 | 0.048 | 0.385 |
| NM_001012304 | *rbm39a* | RNA binding motif protein 39a | 1.51 | 0.007 | 0.168 |
| NM_001044911 | *nrg1* | neuregulin 1 | 1.51 | 0.012 | 0.222 |
| NM_001007032 | *ncor2* | nuclear receptor co-repressor 2 | 1.51 | 0.042 | 0.371 |
| NM_213442 | *srrm1* | serine/arginine repetitive matrix 1 | 1.50 | 0.044 | 0.380 |
| NM_001089366 | *morc3a* | MORC family CW-type zinc finger 3a | 1.50 | 0.048 | 0.385 |
| NM_200595 | *fbxo45* | F-box protein 45 | 1.50 | 0.046 | 0.382 |
| NM_001114926 | *c3orf58b* | C3orf58 chromosome 3 open reading frame 58, b | 1.49 | 0.019 | 0.269 |
| NM_199672 | *stk3* | serine/threonine kinase 3 (STE20 homolog, yeast) | 1.49 | 0.033 | 0.341 |

| Genbank accession number | Gene symbol | Gene description | fold change | P value | Q value |
|---|---|---|---|---|---|
| NM_001020660 | *cyb5r4* | cytochrome b5 reductase 4 | 1.49 | 0.023 | 0.293 |
| NM_001004553 | *ppp3r1b* | protein phosphatase 3 (formerly 2B), regulatory s1ubunit B, alpha isoform, b | 1.49 | 0.010 | 0.201 |
| NM_131183 | *nr2f2* | nuclear receptor subfamily 2, group F, member 2 | 1.48 | 0.026 | 0.308 |
| NM_213489 | *prpf38b* | PRP38 pre-mRNA processing factor 38 (yeast) domain containing B | 1.48 | 0.043 | 0.374 |
| NM_131411 | *aanat2* | arylalkylamine N-acetyltransferase | 1.48 | 0.012 | 0.219 |
| NM_194362 | *ube4b* | ubiquitination factor E4B, UFD2 homolog (S. cerevisiae) | 1.48 | 0.018 | 0.267 |
| NM_194427 | *slc7a4* | solute carrier family 7 (cationic amino acid transporter, y+ system), member 4 | 1.48 | 0.046 | 0.382 |
| NM_001002681 | zgc:86841 | zgc:86841 | 1.47 | 0.045 | 0.382 |
| NM_001024440 | *spc24* | SPC24, NDC80 kinetochore complex component, homolog (S. cerevisiae) | 1.47 | 0.044 | 0.379 |
| NM_001246660 | *rpgrip1l* | RPGRIP1-like | 1.47 | 0.048 | 0.385 |
| NM_001197074 | *u2surp* | U2 snRNP-associated SURP domain containing | 1.47 | 0.022 | 0.284 |
| NM_001110371 | *ppp4r2b* | protein phosphatase 4, regulatory subunit 2b | 1.47 | 0.042 | 0.372 |
| NM_001044914 | *trim8* | tripartite motif-containing 8 | 1.47 | 0.031 | 0.328 |
| NM_213379 | *got2a* | glutamic-oxaloacetic transaminase 2a, mitochondrial (aspartate aminotransferase 2) | 1.46 | 0.014 | 0.245 |
| NM_001082840 | *prkacaa* | protein kinase, cAMP-dependent, catalytic, alpha, genome duplicate a | 1.46 | 0.037 | 0.353 |
| NM_001089401 | *ubtd1a* | ubiquitin domain containing 1a | 1.46 | 0.030 | 0.325 |
| NM_131406 | *raraa* | retinoic acid receptor, alpha a | 1.46 | 0.047 | 0.382 |
| NM_199273 | zgc:66433 | zgc:66433 | 1.45 | 0.012 | 0.228 |
| NM_001045073 | *hsp90aa1.2* | heat shock protein 90, alpha (cytosolic), class A member 1, tandem duplicate 2 | 1.45 | 0.020 | 0.278 |
| NM_200697 | *ckmt2* | creatine kinase, mitochondrial 2 (sarcomeric) | 1.45 | 0.032 | 0.336 |
| NM_131031 | *actb1* | actin, beta 1 | 1.45 | 0.027 | 0.313 |
| NM_001029966 | *trnau1apl* | tRNA selenocysteine 1 associated protein 1, like | 1.44 | 0.041 | 0.370 |
| NM_173274 | *dag1* | dystroglycan 1 | 1.44 | 0.035 | 0.349 |

| Genbank accession number | Gene symbol | Gene description | fold change | P value | Q value |
|---|---|---|---|---|---|
| NM_001007152 | *dpf2* | D4, zinc and double PHD fingers family 2 | 1.44 | 0.046 | 0.382 |
| NM_001030116 | *scrn2* | secernin 2 | 1.44 | 0.036 | 0.351 |
| NM_200472 | *pygmb* | phosphorylase, glycogen (muscle) b | 1.44 | 0.020 | 0.275 |
| NM_001044982 | *cited4a* | Cbp/p300-interacting transactivator, with Glu/Asp-rich carboxy-terminal domain, 4a | 1.44 | 0.029 | 0.322 |
| NM_200867 | *srsf5a* | serine/arginine-rich splicing factor 5a | 1.44 | 0.044 | 0.377 |
| NM_212644 | *dennd5b* | DENN/MADD domain containing 5B | 1.44 | 0.026 | 0.308 |
| NM_001045108 | *fam65a* | family with sequence similarity 65, member A | 1.43 | 0.034 | 0.345 |
| NM_001128539 | *zbtb10* | zinc finger and BTB domain containing 10 | 1.43 | 0.025 | 0.304 |
| NM_199983 | *mylipa* | myosin regulatory light chain interacting protein a | 1.43 | 0.028 | 0.314 |
| NM_131015 | *mafba* | glutamate receptor, ionotropic, N-methyl D-aspartate 1b | 1.43 | 0.026 | 0.309 |
| NM_001017618 | *icmt* | isoprenylcysteine carboxyl methyltransferase | 1.42 | 0.031 | 0.332 |
| NM_001045049 | si:ch211-69g19.2 | si:ch211-69g19.2 | 1.42 | 0.050 | 0.388 |
| NM_212749 | *rhoab* | ras homolog gene family, member Ab | 1.42 | 0.038 | 0.356 |
| NM_199333 | *pkma* | pyruvate kinase, muscle, a | 1.42 | 0.028 | 0.318 |
| NM_001007287 | *fhla* | four and a half LIM domains a | 1.42 | 0.027 | 0.314 |
| NM_001089363 | *ccnl1a* | cyclin L1a | 1.41 | 0.040 | 0.365 |
| NM_213397 | *pfkfb3* | 6-phosphofructo-2-kinase/fructose-2,6-biphosphatase 3 | 1.41 | 0.046 | 0.382 |
| NM_001003612 | *boka* | BCL2-related ovarian killer a | 1.40 | 0.026 | 0.308 |
| NM_001004552 | *pdxdc1* | pyridoxal-dependent decarboxylase domain containing 1 | 1.40 | 0.027 | 0.313 |
| NM_213333 | *sltm* | SAFB-like, transcription modulator | 1.39 | 0.034 | 0.344 |
| NM_200704 | *aanat1* | arylalkylamine N-acetyltransferase 1 | 1.39 | 0.042 | 0.373 |
| NM_001044993 | *nfs1* | NFS1 nitrogen fixation 1 (S. cerevisiae) | 1.38 | 0.041 | 0.370 |
| NM_207072 | *clasp2* | cytoplasmic linker associated protein 2 | 1.38 | 0.041 | 0.369 |

| Genbank accession number | Gene symbol | Gene description | fold change | P value | Q value |
|---|---|---|---|---|---|
| NM_001007295 | zgc:92107 | zgc:92107 | 1.36 | 0.039 | 0.361 |
| NM_001005402 | *cdhr1a* | cadherin-related family member 1a | 1.36 | 0.041 | 0.370 |
| NM_205762 | *traf4a* | tnf receptor-associated factor 4a | 1.36 | 0.049 | 0.386 |
| NM_200150 | *ankrd12* | ankyrin repeat domain 12 | 1.36 | 0.045 | 0.382 |
| NM_001123253 | LOC560226 | novel protein similar to vertebrate breakpoint cluster region (BCR) | 1.35 | 0.044 | 0.377 |

Values were calculated with Cuffdiff, n=3.

Q-values represent adjusted P values with Benjemini and Hochberg procedure for multiple testing.

# Supplementary Table S2

Down-regulated genes in the ES1-expressing rods.  Genes were sorted in ascending order according to the value of fold change.

| Genbank accession number | Gene symbol | Gene description | fold change | P value | Q value |
|---|---|---|---|---|---|
| NM_001256251 | *msnb* | moesin b | 0.08 | 5.0E-5 | 0.005 |
| NM_001003987 | *epb41l3a* | erythrocyte membrane protein band 4.1-like 3a | 0.16 | 2.5E-4 | 0.017 |
| NM_001168350 | *adcy1b* | adenylate cyclase 1b | 0.17 | 0.015 | 0.249 |
| NM_001128759 | zgc:194989 | zgc:194989 | 0.20 | 0.002 | 0.066 |
| NM_001076593 | zgc:153394 | zgc:153394 | 0.20 | 0.003 | 0.097 |
| NM_001017846 | *rgs16* | regulator of G-protein signaling 16 | 0.21 | 0.006 | 0.151 |
| NM_001003629 | *rgs3* | regulator of G-protein signaling 3 | 0.21 | 4.5E-4 | 0.026 |
| NM_001004671 | *uxt* | ubiquitously-expressed transcript | 0.26 | 0.012 | 0.226 |
| NM_212680 | *hmga2* | high mobility group AT-hook 2 | 0.29 | 5.0E-5 | 0.005 |
| NM_213398 | *vamp1* | vesicle-associated membrane protein 1 | 0.30 | 0.042 | 0.372 |
| NM_131098 | *apoeb* | apolipoprotein Eb | 0.30 | 5.0E-5 | 0.005 |
| NM_001025464 | *pdcb* | phosducin b | 0.31 | 5.0E-5 | 0.005 |
| NM_001113589 | *hsp70l* | heat shock cognate 70-kd protein, like | 0.32 | 5.0E-5 | 0.005 |
| NM_001045173 | *ndrg4* | N-myc downstream regulated gene 4 | 0.33 | 5.0E-5 | 0.005 |
| NM_001005924 | *tmem56b* | transmembrane protein 56b | 0.34 | 5.0E-5 | 0.005 |
| NM_200199 | *smu1* | smu-1 suppressor of mec-8 and unc-52 homolog (C. elegans) | 0.35 | 2.5E-4 | 0.017 |
| NR_029934 | *mir204-1* | microRNA 204-1 | 0.35 | 3.0E-4 | 0.019 |
| NM_131397 | *hsp70* | heat shock cognate 70-kd protein | 0.35 | 5.0E-5 | 0.005 |
| NM_001007351 | *btg3* | B-cell translocation gene 3 | 0.35 | 0.007 | 0.170 |
| NM_001020550 | *tp53rk* | TP53 regulating kinase | 0.36 | 0.003 | 0.108 |
| NM_200455 | zgc:63568 | zgc:63568 | 0.37 | 0.013 | 0.236 |

| Genbank accession number | Gene symbol | Gene description | fold change | P value | Q value |
|---|---|---|---|---|---|
| NM_001083843 | *comtb* | catechol-O-methyltransferase b | 0.37 | 5.0E-5 | 0.005 |
| NM_001045138 | *pdcd2* | programmed cell death 2 | 0.38 | 0.012 | 0.223 |
| NM_001034968 | *slit1a* | slit homolog 1a (Drosophila) | 0.38 | 5.0E-5 | 0.005 |
| NM_001007282 | *gpx4a* | glutathione peroxidase 4a | 0.38 | 1.0E-4 | 0.008 |
| NM_201093 | *tnni2a.2* | troponin I, skeletal, fast 2a.2 | 0.38 | 3.0E-4 | 0.019 |
| NM_199569 | *eif2s1l* | eukaryotic translation initiation factor 2, subunit 1 alpha, like | 0.38 | 0.007 | 0.163 |
| NM_001080020 | *grma* | glutamate receptor, metabotropic a | 0.38 | 5.0E-5 | 0.005 |
| NM_001089391 | *slc5a5* | solute carrier family 5 (sodium iodide symporter), member 5 | 0.39 | 2.5E-4 | 0.017 |
| NM_001076667 | *dync1i2a* | dynein, cytoplasmic 1, intermediate chain 2a | 0.40 | 0.048 | 0.385 |
| NM_152961 | *fabp3* | fatty acid binding protein 3, muscle and heart | 0.40 | 5.5E-4 | 0.030 |
| NM_001144131 | *grin1b* | glutamate receptor, ionotropic, N-methyl D-aspartate 1b | 0.40 | 1.0E-4 | 0.008 |
| NM_131869 | *gnat2* | guanine nucleotide binding protein (G protein), alpha transducing activity polypeptide 2 | 0.41 | 5.0E-5 | 0.005 |
| NM_001006093 | *scp2b* | sterol carrier protein 2b | 0.41 | 9.0E-4 | 0.044 |
| NM_001002875 | *gnl3l* | guanine nucleotide binding protein-like 3 (nucleolar)-like | 0.41 | 0.010 | 0.200 |
| NM_131055 | *flh* | floating head | 0.41 | 0.001 | 0.051 |
| NM_213373 | *znf330* | zinc finger protein 330 | 0.43 | 0.018 | 0.267 |
| NM_001003516 | zgc:100908 | zgc:100908 | 0.43 | 0.041 | 0.371 |
| NM_200871 | *pde6c* | phosphodiesterase 6C, cGMP-specific, cone, alpha prime | 0.43 | 2.0E-4 | 0.015 |
| NM_200698 | *cabp5a* | calcium binding protein 5a | 0.43 | 0.003 | 0.108 |
| NM_173284 | *tsr2* | TSR2, 20S rRNA accumulation, homolog (S. cerevisiae) | 0.44 | 0.026 | 0.306 |
| NM_131070 | *mdka* | midkine-related growth factor | 0.44 | 0.005 | 0.138 |
| NM_201072 | *rtn3* | reticulon 3 | 0.44 | 5.5E-4 | 0.030 |
| NM_205645 | *pigf* | phosphatidylinositol glycan, class F | 0.45 | 0.022 | 0.287 |

| Genbank accession number | Gene symbol | Gene description | fold change | P value | Q value |
|---|---|---|---|---|---|
| NM_001089466 | *srebf2* | sterol regulatory element binding transcription factor 2 | 0.45 | 5.0E-5 | 0.005 |
| NM_001004529 | *echs1* | enoyl Coenzyme A hydratase, short chain, 1, mitochondrial | 0.45 | 0.001 | 0.062 |
| NM_001007329 | *slc7a3a* | solute carrier family 7 (cationic amino acid transporter, y+ system), member 3a | 0.45 | 5.0E-5 | 0.005 |
| NM_201059 | *waslb* | Wiskott-Aldrich syndrome-like b | 0.46 | 3.5E-4 | 0.021 |
| NM_001045078 | zk13a21.14 | coiled-coil domain containing 120-like | 0.46 | 0.003 | 0.098 |
| NM_001130612 | *atxn10* | ataxin 10 | 0.46 | 0.019 | 0.272 |
| NM_001037112 | zgc:123238 | zgc:123238 | 0.46 | 0.007 | 0.163 |
| NM_198374 | *pik3ip1* | phosphoinositide-3-kinase interacting protein 1 | 0.46 | 0.003 | 0.098 |
| NM_205653 | *znf410* | zinc finger protein 410 | 0.46 | 0.009 | 0.189 |
| NM_200837 | zgc:73359 | zgc:73359 | 0.46 | 0.033 | 0.340 |
| NM_001115051 | si:dkey-5i22.6 | si:dkey-5i22.6 | 0.47 | 0.006 | 0.150 |
| NM_001020696 | *alg3* | asparagine-linked glycosylation 3 homolog (S. cerevisiae, alpha-1,3-mannosyltransferase) | 0.47 | 0.018 | 0.267 |
| NM_001033749 | *sagb* | S-antigen; retina and pineal gland (arrestin) b | 0.47 | 0.008 | 0.176 |
| NM_198357 | *tcerg1a* | transcription elongation regulator 1a (CA150) | 0.48 | 5.0E-5 | 0.005 |
| NM_001144792 | *cept1a* | choline/ethanolamine phosphotransferase 1a | 0.48 | 0.003 | 0.109 |
| NM_200692 | *ndrg1b* | N-myc downstream regulated gene 1b | 0.48 | 0.006 | 0.153 |
| NM_001077611 | *dars* | aspartyl-tRNA synthetase | 0.48 | 0.005 | 0.137 |
| NM_001002094 | *msrb3* | methionine sulfoxide reductase B3 | 0.48 | 0.006 | 0.154 |
| NM_001025502 | *h2afy2* | H2A histone family, member Y2 | 0.48 | 0.008 | 0.182 |
| NM_001044856 | *ociad2* | OCIA domain containing 2 | 0.48 | 2.5E-4 | 0.017 |
| NM_001037239 | zgc:123177 | zgc:123177 | 0.48 | 0.017 | 0.265 |
| NM_153666 | *tbx24* | T-box 24 | 0.48 | 0.006 | 0.146 |
| NM_001030251 | zgc:114103 | zgc:114103 | 0.49 | 3.5E-4 | 0.021 |

| Genbank accession number | Gene symbol | Gene description | fold change | P value | Q value |
|---|---|---|---|---|---|
| NM_200095 | *asnsd1* | asparagine synthetase domain containing 1 | 0.49 | 0.025 | 0.303 |
| NM_198070 | *tkt* | transketolase | 0.49 | 3.0E-4 | 0.019 |
| NM_001105129 | *srebf1* | sterol regulatory element binding transcription factor 1 | 0.49 | 5.0E-5 | 0.005 |
| NM_200783 | *efna1b* | ephrin A1b | 0.49 | 0.018 | 0.267 |
| NM_001005601 | *tmem18* | transmembrane protein 18 | 0.49 | 0.008 | 0.176 |
| NM_001039808 | *slc2a1a* | solute carrier family 2 (facilitated glucose transporter), member 1a | 0.50 | 9.0E-4 | 0.044 |
| NM_001032366 | *dcun1d4* | DCN1, defective in cullin neddylation 1, domain containing 4 (S. cerevisiae) | 0.50 | 5.0E-5 | 0.005 |
| NM_213126 | *b2ml* | beta-2-microglobulin, like | 0.50 | 5.0E-5 | 0.005 |
| NM_199529 | *mthfd1b* | methylenetetrahydrofolate dehydrogenase (NADP+ dependent) 1b | 0.50 | 0.002 | 0.066 |
| NM_131397 | *hsp70* | heat shock cognate 70-kd protein | 0.51 | 5.0E-5 | 0.005 |
| NM_213205 | *m6pr* | mannose-6-phosphate receptor (cation dependent) | 0.51 | 0.018 | 0.267 |
| NM_001145564 | *cyp2u1* | cytochrome P450, family 2, subfamily U, polypeptide 1 | 0.52 | 0.015 | 0.251 |
| NM_199727 | *serinc1* | serine incorporator 1 | 0.52 | 5.0E-5 | 0.005 |
| NM_205700 | *cfl2* | cofilin 2 (muscle) | 0.52 | 0.009 | 0.198 |
| NM_001003566 | *phpt1* | phosphohistidine phosphatase 1 | 0.52 | 0.009 | 0.198 |
| NM_001045293 | *flj11011l* | hypothetical protein FLJ11011-like (H. sapiens) | 0.52 | 0.006 | 0.163 |
| NM_001004530 | *fuom* | fucose mutarotase | 0.52 | 0.039 | 0.364 |
| NM_001126485 | LOC100006564 | novel NACHT domain containing protein | 0.53 | 0.020 | 0.274 |
| NM_199745 | *esf1* | ESF1, nucleolar pre-rRNA processing protein, homolog (S. cerevisiae) | 0.53 | 0.022 | 0.283 |
| NM_213202 | *gnb3b* | guanine nucleotide binding protein (G protein), beta polypeptide 3b | 0.53 | 0.001 | 0.062 |
| NM_001076569 | zgc:153980 | zgc:153980 | 0.53 | 0.002 | 0.076 |
| NM_213527 | *hiat1b* | hippocampus abundant transcript 1b | 0.53 | 0.003 | 0.087 |
| NM_213104 | *pgrmc2* | progesterone receptor membrane component 2 | 0.53 | 0.006 | 0.156 |

| Genbank accession number | Gene symbol | Gene description | fold change | P value | Q value |
|---|---|---|---|---|---|
| NM_201464 | *impdh2* | IMP (inosine monophosphate) dehydrogenase 2 | 0.54 | 0.025 | 0.303 |
| NM_131205 | *etv5b* | ets variant 5b | 0.54 | 0.006 | 0.155 |
| NM_198368 | *taf12* | TAF12 RNA polymerase II, TATA box binding protein (TBP)-associated factor | 0.54 | 0.018 | 0.267 |
| NM_001020733 | *lin7b* | lin-7 homolog B (C. elegans) | 0.54 | 0.025 | 0.301 |
| NM_178297 | *sepp1a* | selenoprotein P, plasma, 1a | 0.54 | 0.002 | 0.064 |
| NM_001171587 | *prox1b* | prospero-related homeobox gene 1b | 0.54 | 0.008 | 0.176 |
| NM_001115122 | *dpp6b* | dipeptidyl-peptidase 6b | 0.55 | 0.002 | 0.082 |
| NM_200745 | *edf1* | endothelial differentiation-related factor 1 | 0.55 | 0.026 | 0.308 |
| NM_205570 | zgc:73226 | zgc:73226 | 0.55 | 2.5E-4 | 0.017 |
| NM_205759 | *eepd1* | endonuclease/exonuclease/phosphatase family domain containing 1 | 0.55 | 0.032 | 0.336 |
| NM_001034988 | *cx41.8* | connexin 41.8 | 0.55 | 0.018 | 0.267 |
| NM_001089338 | zgc:162958 | zgc:162958 | 0.55 | 0.010 | 0.208 |
| NM_001007422 | zgc:100824 | zgc:100824 | 0.56 | 0.038 | 0.356 |
| NM_001023581 | *tmem167a* | transmembrane protein 167A | 0.56 | 0.021 | 0.281 |
| NM_001014313 | *tmem35* | transmembrane protein 35 | 0.56 | 0.005 | 0.131 |
| NM_001114702 | zgc:174710 | zgc:174710 | 0.56 | 1.1E-3 | 0.050 |
| NM_001083544 | *rbx1* | ring-box 1 | 0.56 | 0.010 | 0.207 |
| NM_001190382 | *sell* | selenoprotein L | 0.57 | 0.040 | 0.365 |
| NM_131400 | *tefa* | thyrotroph embryonic factor a | 0.57 | 0.002 | 0.066 |
| NM_212629 | *ubr7* | ubiquitin protein ligase E3 component n-recognin 7 | 0.57 | 0.007 | 0.163 |
| NM_199582 | *tpd52l2b* | tumor protein D52-like 2b | 0.57 | 0.046 | 0.382 |
| NM_199869 | *insig1* | insulin induced gene 1 | 0.57 | 0.041 | 0.370 |
| NM_001005990 | *kansl2* | KAT8 regulatory NSL complex subunit 2 | 0.57 | 0.043 | 0.375 |

| Genbank accession number | Gene symbol | Gene description | fold change | P value | Q value |
|---|---|---|---|---|---|
| NM_152886 | *appb* | amyloid beta (A4) precursor protein b | 0.58 | 4.0E-4 | 0.024 |
| NM_212990 | *eif3i* | eukaryotic translation initiation factor 3, subunit I | 0.58 | 0.036 | 0.352 |
| NM_001039981 | zgc:136866 | zgc:136866 | 0.58 | 0.047 | 0.382 |
| NM_001007291 | *oxct1a* | 3-oxoacid CoA transferase 1a | 0.58 | 0.030 | 0.325 |
| NM_001082997 | *si:dkey-23c22.6* | si:dkey-23c22.6 | 0.58 | 0.003 | 0.097 |
| NM_001004648 | *nudt4b* | nudix (nucleoside diphosphate linked moiety X)-type motif 4b | 0.58 | 0.018 | 0.267 |
| NM_200766 | *paip2b* | poly(A) binding protein interacting protein 2B | 0.58 | 0.011 | 0.218 |
| NM_001020775 | zgc:113201 | zgc:113201 | 0.59 | 0.050 | 0.389 |
| NM_001077391 | zgc:153997 | zgc:153997 | 0.59 | 0.002 | 0.084 |
| NM_001089335 | *bri3bp* | bri3 binding protein | 0.59 | 0.023 | 0.290 |
| NM_001002634 | *nipal3* | NIPA-like domain containing 3 | 0.59 | 0.028 | 0.314 |
| NM_199793 | *dazap2* | DAZ associated protein 2 | 0.59 | 0.003 | 0.095 |
| NM_001006087 | *ormdl3* | ORM1-like 3 (S. cerevisiae) | 0.59 | 0.007 | 0.163 |
| NM_199481 | *ccng1* | cyclin G1 | 0.59 | 0.009 | 0.196 |
| NM_212575 | *mtx2* | metaxin 2 | 0.59 | 0.035 | 0.346 |
| NM_001079969 | *spoplb* | speckle-type POZ protein-like b | 0.60 | 0.040 | 0.365 |
| NM_201036 | *vti1b* | vesicle transport through interaction with t-SNAREs homolog 1B (yeast) | 0.60 | 0.027 | 0.312 |
| NM_001002707 | zgc:92606 | zgc:92606 | 0.60 | 0.005 | 0.139 |
| NM_001004605 | *arrdc3b* | arrestin domain containing 3b | 0.60 | 0.006 | 0.156 |
| NM_201067 | *rorab* | RAR-related orphan receptor A, paralog b | 0.60 | 0.010 | 0.203 |
| NM_214819 | *rcan2* | regulator of calcineurin 2 | 0.60 | 0.017 | 0.265 |
| NM_001020714 | zgc:113162 | zgc:113162 | 0.60 | 0.048 | 0.385 |
| NM_198978 | *pdcd4b* | programmed cell death 4b | 0.60 | 0.004 | 0.120 |

| Genbank accession number | Gene symbol | Gene description | fold change | P value | Q value |
|---|---|---|---|---|---|
| NM_214770 | *rab11ba* | RAB11B, member RAS oncogene family, a | 0.60 | 0.038 | 0.356 |
| NM_001005932 | *tars* | threonyl-tRNA synthetase | 0.60 | 0.030 | 0.325 |
| NM_001080079 | *mapkapk3* | mitogen-activated protein kinase-activated protein kinase 3 | 0.60 | 0.015 | 0.249 |
| NM_001024221 | *wdr33* | WD repeat domain 33 | 0.60 | 0.045 | 0.381 |
| NM_001045564 | *psmb7* | proteasome (prosome, macropain) subunit, beta type, 7 | 0.60 | 0.039 | 0.364 |
| NM_213407 | *zranb2* | zinc finger, RAN-binding domain containing 2 | 0.61 | 0.006 | 0.146 |
| NM_001014306 | *lifra* | leukemia inhibitory factor receptor alpha a | 0.61 | 0.009 | 0.196 |
| NM_199681 | *phb2* | prohibitin 2 | 0.61 | 0.015 | 0.251 |
| NM_212708 | *slc16a3* | solute carrier family 16 (monocarboxylic acid transporters), member 3 | 0.61 | 0.006 | 0.156 |
| NM_001017732 | *prkar1ab* | protein kinase, cAMP-dependent, regulatory, type I, alpha (tissue specific extinguisher 1) b | 0.61 | 0.007 | 0.163 |
| NM_212850 | *glcea* | glucuronyl C5-epimerase a | 0.61 | 0.022 | 0.283 |
| NM_001003875 | *snrnp70* | small nuclear ribonucleoprotein 70 (U1) | 0.61 | 0.002 | 0.066 |
| NM_205745 | *wdfy2* | WD repeat and FYVE domain containing 2 | 0.61 | 0.046 | 0.382 |
| NM_201171 | *dusp4* | dual specificity phosphatase 4 | 0.62 | 0.008 | 0.176 |
| NM_131065 | *nr1d2b* | nuclear receptor subfamily 1, group D, member 2b | 0.62 | 0.025 | 0.304 |
| NM_201111 | *hook2* | hook homolog 2 (Drosophila) | 0.62 | 0.009 | 0.196 |
| NM_001044840 | *tmem181* | transmembrane protein 181 | 0.62 | 0.024 | 0.297 |
| NM_001029968 | *nif3l1* | NIF3 NGG1 interacting factor 3-like 1 (S. cerevisiae) | 0.62 | 0.037 | 0.353 |
| NM_001025538 | *tspan3a* | tetraspanin 3a | 0.62 | 0.002 | 0.069 |
| NM_001256646 | *flrt3* | fibronectin leucine rich transmembrane 3 | 0.62 | 0.010 | 0.209 |
| NM_001080074 | zgc:158316 | zgc:158316 | 0.62 | 0.027 | 0.312 |
| NM_213435 | zgc:55683 | zgc:55683 | 0.63 | 0.033 | 0.337 |
| NM_131758 | *stom* | stomatin | 0.63 | 0.005 | 0.137 |

| Genbank accession number | Gene symbol | Gene description | fold change | P value | Q value |
|---|---|---|---|---|---|
| NM_001013312 | zgc:113324 | zgc:113324 | 0.63 | 0.042 | 0.374 |
| NM_001128811 | zgc:194176 | zgc:194176 | 0.63 | 0.024 | 0.300 |
| NM_001201344 | *slc43a3a* | solute carrier family 43, member 3a | 0.63 | 0.007 | 0.163 |
| NM_212904 | *chmp2bb* | chromatin modifying protein 2Bb | 0.63 | 0.046 | 0.382 |
| NM_001110479 | *trpm1b* | transient receptor potential cation channel, subfamily M, member 1b | 0.63 | 0.021 | 0.281 |
| NM_001089562 | *rab8a* | RAB8A, member RAS oncogene family | 0.63 | 0.020 | 0.278 |
| NM_001089384 | zgc:162698 | zgc:162698 | 0.63 | 0.009 | 0.191 |
| NM_001003731 | zgc:91910 | zgc:91910 | 0.63 | 0.048 | 0.385 |
| NM_001045295 | *farsa* | phenylalanyl-tRNA synthetase, alpha subunit | 0.64 | 0.047 | 0.382 |
| NM_213308 | *stk25b* | serine/threonine kinase 25b | 0.64 | 0.019 | 0.269 |
| NM_001007424 | *dhrs13a.3* | dehydrogenase/reductase (SDR family) member 13a.3 | 0.64 | 0.032 | 0.336 |
| NM_001003623 | *naa50* | N(alpha)-acetyltransferase 50, NatE catalytic subunit | 0.64 | 0.039 | 0.361 |
| NM_001044942 | *parp1* | poly (ADP-ribose) polymerase family, member 1 | 0.64 | 0.027 | 0.312 |
| NM_199919 | *ap2b1* | adaptor-related protein complex 2, beta 1 subunit | 0.65 | 0.013 | 0.234 |
| NM_213262 | *eif5a2* | eukaryotic translation initiation factor 5A2 | 0.65 | 0.008 | 0.174 |
| NM_001079977 | *hmgcra* | 3-hydroxy-3-methylglutaryl-Coenzyme A reductase a | 0.65 | 0.009 | 0.191 |
| NM_182967 | *calm3a* | calmodulin 3a (phosphorylase kinase, delta) | 0.65 | 0.014 | 0.244 |
| NM_001008617 | *ttc26* | tetratricopeptide repeat domain 26 | 0.65 | 0.040 | 0.369 |
| NM_213087 | *sdf4* | stromal cell derived factor 4 | 0.65 | 0.013 | 0.228 |
| NM_201327 | *ssr1* | signal sequence receptor, alpha | 0.65 | 0.030 | 0.328 |
| NM_001170599 | *tbx21* | T-box 21 | 0.65 | 0.030 | 0.328 |
| NM_001037699 | *golph3l* | golgi phosphoprotein 3-like | 0.65 | 0.048 | 0.385 |
| NM_199555 | *hmgb1a* | high-mobility group box 1a | 0.65 | 0.011 | 0.214 |

| Genbank accession number | Gene symbol | Gene description | fold change | P value | Q value |
|---|---|---|---|---|---|
| NM_001003861 | *rpl9* | ribosomal protein L9 | 0.66 | 0.017 | 0.264 |
| NM_131451 | *irbp* | interphotoreceptor retinoid-binding protein | 0.66 | 0.030 | 0.325 |
| NM_199965 | *rbp4l* | retinol binding protein 4, like | 0.66 | 0.019 | 0.271 |
| NM_001040313 | zgc:136739 | zgc:136739 | 0.66 | 0.017 | 0.266 |
| NM_001082872 | *nat15* | N-acetyltransferase 15 (GCN5-related, putative) | 0.66 | 0.048 | 0.385 |
| NM_001076663 | *rabggta* | Rab geranylgeranyltransferase, alpha subunit | 0.66 | 0.045 | 0.381 |
| NM_001128358 | LOC797250 | novel protein similar to H.sapiens solute carrier family 7 (cationic amino acid transporter, y+ system), member 5 | 0.66 | 0.011 | 0.216 |
| NM_001083002 | *arid3b* | AT rich interactive domain 3B (Bright like) | 0.66 | 0.012 | 0.219 |
| NM_001030102 | *brms1la* | breast cancer metastasis-suppressor 1-like a | 0.67 | 0.040 | 0.365 |
| NM_001103111 | *mntb* | MAX binding protein b | 0.67 | 0.027 | 0.312 |
| NM_194417 | *sepw2a* | selenoprotein W, 2a | 0.67 | 0.011 | 0.215 |
| NM_213529 | *tmem50a* | transmembrane protein 50A | 0.67 | 0.040 | 0.367 |
| NM_001045402 | *cbx3a* | chromobox homolog 3a (HP1 gamma homolog, Drosophila) | 0.67 | 0.048 | 0.385 |
| NM_001114585 | zgc:174944 | zgc:174944 | 0.67 | 0.020 | 0.274 |
| NM_173254 | *atp6v1e1b* | ATPase, H+ transporting, lysosomal, V1 subunit E isoform 1b | 0.67 | 0.015 | 0.251 |
| NM_001013337 | zgc:113200 | zgc:113200 | 0.67 | 0.029 | 0.319 |
| NM_001045052 | *tmem106bb* | transmembrane protein 106Bb | 0.67 | 0.013 | 0.231 |
| NM_131710 | *ctsd* | cathepsin D | 0.68 | 0.024 | 0.296 |
| NM_001130192 | *pnrc2* | proline-rich nuclear receptor coactivator 2 | 0.68 | 0.020 | 0.273 |
| NM_212912 | *tmed7* | transmembrane emp24 protein transport domain containing 7 | 0.68 | 0.029 | 0.319 |
| NM_001077376 | si:ch211-133n4.4 | si:ch211-133n4.4 | 0.68 | 0.023 | 0.290 |
| NM_131465 | *pcmt* | l-isoaspartyl protein carboxyl methyltransferase | 0.69 | 0.023 | 0.291 |
| NM_001002353 | *dnajb6a* | DnaJ (Hsp40) homolog, subfamily B, member 6a | 0.69 | 0.045 | 0.381 |

| Genbank accession number | Gene symbol | Gene description | fold change | P value | Q value |
|---|---|---|---|---|---|
| NM_001077568 | *ctsf* | cathepsin F | 0.69 | 0.016 | 0.256 |
| NM_201166 | *dctn2* | dynactin 2 (p50) | 0.69 | 0.037 | 0.356 |
| NM_178306 | *tph1a* | tryptophan hydroxylase 1 (tryptophan 5-monooxygenase) a | 0.69 | 0.028 | 0.314 |
| NM_200009 | *eef1a1a* | eukaryotic translation elongation factor 1 alpha 1a | 0.69 | 0.015 | 0.248 |
| NM_199666 | *ndfip1* | Nedd4 family interacting protein 1 | 0.70 | 0.019 | 0.271 |
| NM_200362 | *tmem106ba* | transmembrane protein 106Ba | 0.70 | 0.029 | 0.320 |
| NM_001025475 | *idi1* | isopentenyl-diphosphate delta isomerase 1 | 0.70 | 0.041 | 0.371 |
| NM_001099448 | *wwp2* | WW domain containing E3 ubiquitin protein ligase 2 | 0.70 | 0.048 | 0.385 |
| NM_001144040 | *cog3* | component of oligomeric golgi complex 3 | 0.70 | 0.038 | 0.356 |
| NM_199957 | *ppiab* | peptidylprolyl isomerase Ab (cyclophilin A) | 0.70 | 0.050 | 0.389 |
| NM_001167570 | *pvrl1a* | poliovirus receptor-related 1a | 0.70 | 0.050 | 0.389 |
| NM_200925 | *stau2* | staufen, RNA binding protein, homolog 2 (Drosophila) | 0.71 | 0.035 | 0.350 |
| NM_201112 | *arl6ip1* | ADP-ribosylation factor-like 6 interacting protein 1 | 0.71 | 0.031 | 0.332 |
| NM_178290 | *selt1a* | selenoprotein T, 1a | 0.71 | 0.034 | 0.346 |
| NM_001164027 | *npepps* | aminopeptidase puromycin sensitive | 0.71 | 0.037 | 0.356 |
| NM_001252649 | si:ch211-117m20.5 | si:ch211-117m20.5 | 0.72 | 0.037 | 0.353 |
| NM_213250 | *csnk1db* | casein kinase 1, delta b | 0.73 | 0.048 | 0.385 |
| NM_199587 | *lztfl1* | leucine zipper transcription factor-like 1 | 0.73 | 0.049 | 0.387 |
| NM_001002298 | *asna1* | arsA arsenite transporter, ATP-binding, homolog 1 (bacterial) | 0.73 | 0.046 | 0.382 |

Values were calculated with Cuffdiff, n=3.

Q-values represent adjusted P values with Benjemini and Hochberg procedure for multiple testing.

Genes coding cone opsins were excluded because they can be derived from contamination of cones or misalignment of rhodopsin transcripts which are expressed in rods the most abundantly.

**Supplementary Methods**

**Antibodies.**

Antigens used for production of each antibody prepared in our laboratory or raised commercially were purified carp ES1 (AB794660) [4], a peptide fragment of carp mAAT (Ser28-Lys428; AB793727), purified carp rhodopsin, a partial peptide of the sequence conserved between carp red and green opsins (STSKTEVSSVAPA; AB055656.1 and AB110602.2, respectively) [5], and peptide fragments of carp cone-type arrestin-1 and -2 (Arg66-Lys102; AB794065 and Arg68-Ala110; AB794066, respectively) [5]. Each antibody was used at the following dilutions for immunoblotting; anti-ES1 (1:1,000), anti-alpha-tubulin (1:1,000), anti-mAAT (1:1,000), anti-TOM20 (1:1,000), anti-red/green opsins (1:3,000), anti-rhodopsin (1:3,000), anti-AMPKα (1:300) and anti-phospho-AMPKα (1:300), and for immunohistochemistry; anti-ES1 (1:500), anti-mAAT (1:300), anti-TOM20 (1:300) and anti-red/green opsins (1:500) and for immunocytochemistry; anti-cone-type arrestin-1 (1:300) and -2 (1:300).

**Subcellular fractionation**

Cells dissociated from retinas were suspended in a potassium gluconate buffer (115 mM potassium gluconate, 2.5 mM KCl, 2 mM $MgCl_2$, 10 mM HEPES, 0.2 mM EGTA, 0.1 mM $CaCl_2$, pH 7.5). After freeze-thawed three times, the cells were centrifuged at 100,000 x g for 10 min three times and fractionated into supernatant as a soluble fraction and precipitation as a membrane fraction.

***In situ* hybridization.**

*In situ* hybridization was performed using 10-μm frozen ocular sections prepared from light-adapted zebrafish as previously described [6]. Complementary RNA probes against ES1 mRNA at 372-755 bases of open reading frame and rhodopsin mRNA at 718-936 bases were synthesized by using DIG RNA Labeling Kit (Roche Diagnostics K.K.).

**Immunohistochemical studies.**

Eyecups of adult zebrafish were isolated and fixed at a time point of 4 h after light onset (i.e. 4 h light adaptation). And whole embryos were fixed at various time points within 3-10 h after light onset. Immunohistochemistry was performed using 10-μm frozen sections as previously described [6]. Secondary antibodies used are anti-mouse IgG and anti-rabbit IgG which are conjugated with either Alexa Fluor 488

(1:300 dilution, Molecular probes), Alexa Fluor 568 (1:300 dilution, Molecular probes) or ATTO 655 (1:200 dilution, Sigma-Aldrich). Immunostained sections were observed by using a BX-51 fluorescence microscope (Olympus) and a confocal fluorescence microscope (FluoView FV1000, Olympus). Signal intensities in the confocal images were determined by the aid of software imageJ.

**Electron microscopic studies.**

Light conditions before tissue fixations were described above (see **Immunohistochemical studies**). Eyecups from adult zebrafish or whole embryos were fixed with Ringer's solution containing 1% (w/v) paraformaldehyde and 2.5% (w/v) glutaraldehyde for 2 h at 4°C, followed by treatment with 0.5% osmium tetroxide in Ringer’s solution for 30 min at 4°C. After dehydration through a graded ethanol series, the tissue fragments were embedded either in epoxy resin Quetol 812 (Nissin EM) or LR-White (for immunoelectron microscopy, London Resin Company), and were cut into ultrathin sections (80-100 nm) by using an ultramicrotome (EM-Ultracut UCT; Leica). After immunostaining process described in Methods or without the immunostaining, the sections were post-stained with a 4% uranyl acetate solution and a lead staining solution (Sigma-Aldrich), and observed by using a transmission electron microscope (JEM-1011; JEOL).

**Read mapping and estimation of gene expression on RNA-seq data.**

Short reads with less than 40 bases and low-quality reads, more than 30% of which bases showed a Phred score less than 17, were excluded by FastX-Tool kit (http://hannonlab.cshl.edu/fastx_toolkit/), and the remaining reads were mapped to the zebrafish reference genome (Zv9) from Ensembl database (http://www.ensembl.org/Danio_rerio/Info/Index) using TopHat2 software (v2.0.11)[7]. We subsequently quantified gene expression levels by Cufflinks software (v2.2.1) and determined differentially expressed genes by Cuffdiff software[8]. Three replicates for each transgenic line were subjected to these analyses. Expression levels of some important genes were validated by quantitative real-time PCR assays as described in Methods.

**GO and pathway enrichment analyses.**

Based on the Cuffdiff result, more than 1.5 fold up- or down-regulated genes were subjected to GO analysis using WEB-based Gene Stet Analysis Toolkit (WebGestalt) database[9]. Pathway enrichment analysis for these genes was performed by

Wikipathways included in WebGestalt. All genes (10,633 genes) whose expressions were detected in ES1-TG and/or EGFP-TG were used as reference for these statistical analyses.

**Quantification of mtDNA content.**

Retinas were isolated from the ES1-TG and EGFP-TG at a time point of 8 h after light onset (i.e. 8 h light adaptation). Rods composed of the outer segment and the ellipsoid were purified from the retinas as described in "Estimation of cellular energy states of rods" in Methods section. The purified rods were treated with lysis buffer (50 mM Tris–HCl pH 8.5, 1 mM EDTA, 0.5% Tween-20, 200 μg/ml proteinase K) at 55°C for 2 h, and then proteinase K was denatured at 95°C for 10 min. Then DNAs were purified with Fast Gene Gel/PCR Extraction Kit (NIPPON Genetics Co. Ltd., Tokyo, Japan). Mitochondrial DNA contents were quantified by real-time PCR using a primer set for mtDNA encoded NADH dehydrogenase 1 as described in ref. 10 in two technical replicates. The content of mtDNA was normalized by the number of cells.

**References for Supplementary Information**


1. Ma, J. *et al.* A visual pigment expressed in both rod and cone photoreceptors. *Neuron* **32**, 451–461 (2001).
2. Asaoka, Y., Mano, H., Kojima, D. & Fukada, Y. Pineal expression-promoting element (PIPE), a *cis*-acting element, directs pineal-specific gene expression in zebrafish. *Proc. Natl. Acad. Sci. USA* **99**, 15456–15461 (2002).
3. Kim, M. J., Kang, K. H., Kim C. H. & Choi, S. Y. Real-time imaging of mitochondria in transgenic zebrafish expressing mitochondrially targeted GFP. *Biotechniques* **45**, 331–334 (2008).
4. Komatsu, Y., Tachibanaki, S. & Kawamura, S. ES1: an abundant protein specifically expressed in cone inner segments of the carp retina. *Seibutsu Butsuri* 46 (supplement_2): 255 (2006).
5. Tomizuka, J., Tachibanaki, S. & Kawamura, S. Phosphorylation-independent suppression of light-activated visual pigment by arrestin in carp rods and cones. *J. Biol. Chem.* **290**, 9399-9411(2015).
6. Wada, Y., Sugiyama, J., Okano, T. & Fukada, Y. GRK1 and GRK7: Unique cellular distribution and widely different activities of opsin phosphorylation in the zebrafish rods and cones. *J. Neurochem.* **98**, 824–837 (2006).
7. Kim, D. *et al.* TopHat2: accurate alignment of transcriptomes in the presence of

insertions, deletions and gene fusions. *Genome Biol.* **14**, R36 (2013).
8. Trapnell, C. *et al.* Differential gene and transcript expression analysis of RNA-seq experiments with TopHat and Cufflinks. *Nat. Protoc.* **7**, 562–578 (2012).
9. Wang, J. *et al.* WEB-based GEne SeT AnaLysis Toolkit (WebGestalt): update 2013. *Nucleic Acids Res.* **41**, W77–W83 (2013).
10. Artuso, L. *et al.* Mitochondrial DNA metabolism in early development of zebrafish (Danio rerio). *Biochim. Biophys. Acta* **1817**, 1002–1011 (2012).
